# X chromosome dosage and the genetic impact across human tissues

**DOI:** 10.1186/s13073-023-01169-4

**Published:** 2023-03-28

**Authors:** Mette Viuff, Anne Skakkebæk, Emma B. Johannsen, Simon Chang, Steen Bønlykke Pedersen, Katrine Meyer Lauritsen, Mette Glavind Bülow Pedersen, Christian Trolle, Jesper Just, Claus H. Gravholt

**Affiliations:** 1 Department of Molecular Medicine, Aarhus University Hospital, Palle-Juul Jensens Boulevard 99, Aarhus N, 8200 Denmark; 2 Department of Gynecology and Obstetrics, Aarhus University Hospital, Palle-Juul Jensens Boulevard 99, Aarhus N, 8200 Denmark; 3 Department of Clinical Medicine, Aarhus University, Palle-Juul Jensens Boulevard 99, Aarhus N, 8200 Denmark; 4 Department of Clinical Genetics, Aarhus University Hospital, Palle-Juul Jensens Boulevard 99, Aarhus N, 8200 Denmark; 5 Department of Endocrinology and Internal Medicine and Medical Research Laboratories, Aarhus University Hospital, Palle-Juul Jensens Boulevard 99, Aarhus N, 8200 Denmark

**Keywords:** Sex chromosome aneuploidies, DNA methylation, RNA expression, Epigenetics, Genetics

## Abstract

**Background:**

Sex chromosome aneuploidies (SCAs) give rise to a broad range of phenotypic traits and diseases. Previous studies based on peripheral blood samples have suggested the presence of ripple effects, caused by altered X chromosome number, affecting the methylome and transcriptome. Whether these alterations can be connected to disease-specific tissues, and thereby having clinical implication for the phenotype, remains to be elucidated.

**Methods:**

We performed a comprehensive analysis of X chromosome number on the transcriptome and methylome in blood, fat, and muscle tissue from individuals with 45,X, 46,XX, 46,XY, and 47,XXY.

**Results:**

X chromosome number affected the transcriptome and methylome globally across all chromosomes in a tissue-specific manner. Furthermore, 45,X and 47,XXY demonstrated a divergent pattern of gene expression and methylation, with overall gene downregulation and hypomethylation in 45,X and gene upregulation and hypermethylation in 47,XXY. In fat and muscle, a pronounced effect of sex was observed. We identified X chromosomal genes with an expression pattern different from what would be expected based on the number of X and Y chromosomes. Our data also indicate a regulatory function of Y chromosomal genes on X chromosomal genes.

Fourteen X chromosomal genes were downregulated in 45,X and upregulated in 47,XXY, respectively, in all three tissues (*AKAP17A*, *CD99*, *DHRSX*, *EIF2S3*, *GTPBP6*, *JPX*, *KDM6A*, *PP2R3B*, *PUDP*, *SLC25A6*, *TSIX*, *XIST*, *ZBED1*, *ZFX*). These genes may be central in the epigenetic and genomic regulation of sex chromosome aneuploidies.

**Conclusion:**

We highlight a tissue-specific and complex effect of X chromosome number on the transcriptome and methylome, elucidating both shared and non-shared gene-regulatory mechanism between SCAs.

**Supplementary Information:**

The online version contains supplementary material available at 10.1186/s13073-023-01169-4.

## Background

In females, monosomy X results in Turner syndrome (TS; 45,X) and in males an additional X chromosome gives rise to Klinefelter syndrome (KS, 47,XXY) [1, 2]. Both syndromes share overlapping phenotypic traits such as infertility, hypergonadotropic hypgonadism, an increased risk of the metabolic syndrome, type II diabetes, osteoporosis, autoimmune disorders, and neuropsychiatric and neurodevelopmental disorders [3–6], all adding to an increased overall morbidity and mortality [3, 5, 7]. However, syndrome-specific traits are also prevalent in both TS and KS, such as cardiac anomalies in TS and venous thrombosis in KS [8, 9]. The overlapping phenotypic traits, in addition to individual groupwise stigmata, indicates that X chromosome number impacts central biological pathways involved in disease development. Elucidating how X chromosome number impacts the methylome, transcriptome and proteome, and regulates genomic pathways and networks, is essential in deciphering the molecular underpinnings of the phenotype in TS and KS as well as other sex chromosome aneuplodies (SCAs).

During the past decade, several studies have widened the knowledge about sex chromosome biology [10–16]. Collectively, they demonstrate a significant impact of especially X chromosome number on the human transciptome and methylome. These studies have primarily used peripheral blood or cells derived from blood for genomic analysis [10–16]. However, gene expression as well as DNA methylation is tissue-specific. Thus, blood may not always be a representative and relevant tissue to study to elucidate the molecular underpinnings of altered sex chromosome number ultimately leading to the phenotype seen in SCAs. Presently, a significant gap exists as to how sex chromosome number affects the transcriptome and methylome in specific tissues.

**Fig. 1:**
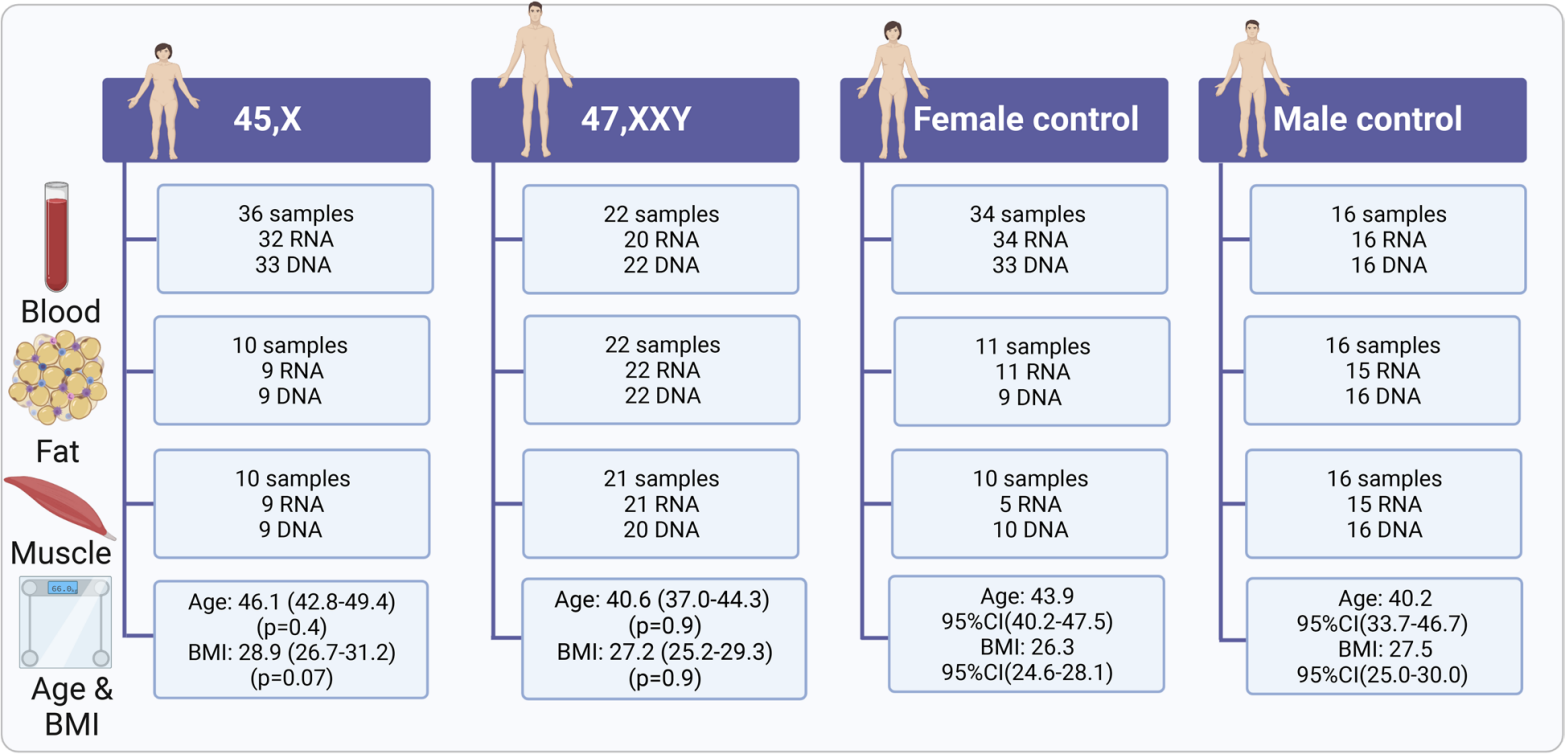
Illustration of the included blood, fat, and muscle samples from 45,X (*n* = 36), 46,XX (*n* = 34), 46,XY (*n* = 16), and 47,XXY (*n* = 22)

To this end, we collected blood samples, fat and muscle tissue biopsies from adult individuals with diverse karyotypes including 45,X (*n* = 36), 46,XX (*n* = 34), 46,XY (*n* = 16) and 47,XXY (*n* = 22) and performed gene expression analysis (RNA-seq) and DNA methylation analysis (850 k- Illumina Infinium assay), allowing comprehensive integrative analysis of the X chromosome number effect on both the transcriptome and methylome across three different target tissues (Fig. 1).

## Methods

### Design

The samples in this study were derived from four cross-sectional studies on patients with TS and KS and controls (NCT00624949, NCT00999310, NCT02526628, NCT01678261). The primary outcomes of the clinical trials have been published [10–14], and the circular RNA profile has been characterized in a subset of these samples [15]. The first participant was enrolled in 2012 and the last was enrolled in 2021. 47,XXY males (*n* = 22, median age (95%CI) 40.6 (37.0–44.3) were recruited through endocrine and clinical genetic hospital outpatient clinics as well as fertility clinics in Denmark, whereas 45,X women (*n* = 36, median age (95%CI) 46.1 (42.8–49.4) were recruited through the Danish National Society of Tuner Syndrome Contact Group and an endocrine outpatient clinic in Denmark. Healthy, age-matched men (*n* = 16, median age (95%CI): 40.2 (33.7–46.7) and women (*n* = 34, median age (95%CI) 43.9 (40.2–47.5) were recruited through advertisement to serve as controls. We obtained blood samples and abdominal subcutaneous fat tissue biopsies from all 22 47,XXY, and vastus lateralis muscle biopsies from 21 47,XXY. Of the 36 patients with TS, all had a blood sample drawn, and 10 had an abdominal subcutaneous fat tissue biopsy and a vastus lateralis muscle biopsy taken. Sixteen male controls contributed with a blood sample, a subcutaneous fat tissue biopsy, and a vastus lateralis muscle biopsy. Of the 34 female controls, all had a blood sample drawn, 11 had an abdominal subcutaneous fat tissue biopsy, and 10 had a vastus lateralis muscle biopsy taken. DNA methylation were not performed on four blood samples (45,X, *n* = 3; female control, *n* = 1), two fat tissue samples (45,X, *n* = 1; female control, *n* = 2), and in one muscle tissue sample (47,XXY, *n* = 1) due to technical reasons. RNA sequencing was not performed on 6 blood samples (45,X, *n* = 4, 47,XXY, *n* = 2), two fat samples (45,X, *n* = 1; male controls, *n* = 1), and 6 muscle tissue samples (45,X. *n* = 1; female controls, *n* = 5, male controls, *n* = 1) due to technical reasons (Fig. 1).

### Sample collection

Blood samples for RNA extraction were drawn from the antecubital vein into PAXgene Blood RNA Tubes, placed 2 h at room temperature, and then stored overnight at − 21°, before storage at − 80°. Blood samples for DNA extraction were drawn into EDTA-treated tubes, and the peripheral blood samples were stored immediately at − 80 °C. Abdominal subcutaneous fat tissue biopsies were obtained from the lower abdomen by liposuction technique. The procedure was performed under sterile conditions and local anesthesia (10 ml lidocaine, 10 mg/ml). The biopsies were immediately cleaned and snap-frozen in liquid nitrogen and stored at − 80°. Muscle biopsies were taken from the vastus lateralis. The procedure was performed under sterile conditions and local anesthesia (10 ml lidocaine, 10 mg/ml). A small incision was made in the skin parallel to the orientation of the muscle fibers below. The muscle tissue was extracted using a 5-mm Bergström needle. The biopsies were immediately snap-frozen in liquid nitrogen and stored at − 80°.

### RNA and DNA extraction

#### Blood

Blood total RNA was purified using the PaxGene blood Kit 262,174 (Qiagen) followed by on-chip electrophoresis on a Tapestation 4200 RNA Screen Tape System (Agilent) and by UV measurements on a Lunatic (Unchained Labs). Synthesis of directional RNA-seq libraries was conducted using the KAPA RNA HyperPrep with RiboErase Globin (HMR) (Roche) following the recommended procedure. Library preparation was automated on a Sciclone NGS (Caliper, Perkin Elmer) liquid handling robot. RNA-seq library quality was estimated by on-chip electrophoresis on a Tapestation 4200 D100 Screen Tape System (Agilent). The concentrations of the libraries were estimated using the Qubit dsDNA HS Assay (Thermo Fisher). Five hundred nanograms of total RNA was used as input.

Genomic DNA from the blood samples was extracted using the QIAmp® Mini Kit (Qiagen, Germany).

#### Fat tissue

The frozen biopsies were dissociated using a CovarisSP02, and DNA and RNA was extracted by the AllPrep DNA/RNA/PROTEIN Mini kit 8004 (Qiagen). Synthesis of directional RNA-seq libraries were conducted as described above using the KAPA RNA HyperPrep with RiboErase kit (HMR) (Roche).

#### Muscle tissue

The biopsies were sliced into 10-µm sections using a cryostat. RNA was extracted from all sections by the RNeasy Fibrouse tissue Mini kit (Qiagen). Synthesis of directional RNA-seq libraries were conducted as described above using the KAPA RNA HyperPrep with RiboErase kit (HMR) (Roche). DNA from muscle tissue was extracted using Gentra Puregene tissue kit catalog number 158667.

The isolated DNA from all tissues was quantified using a Qubit fluorimeter (manufacturer and model).

### RNA sequencing and bioinformatics

The RNA-seq libraries were multiplexed paired-end sequenced on an Illumina NovaSeq 6000 (100 bp). Paired de-multiplexed fastq files were quality controlled using FastQC (Babraham Bioinformatics). Adaptor removal, in addition to trimming of low-quality ends, was then carried out using Trim Galore (Babraham Bioinformatics). Gene expression was quantified by quasi-mapping using Salmon [16]. A decoy-aware transcriptome index was built based on the hg38 transcriptome and selective alignment was run using the fastq pairs as input. Transcript abundancies were summarized to a gene level using the R package Tximeta [17]. Differential expression was then assessed using the R Bioconductor package DESeq2 using adjusted *p*-value < 0.05 (FDR < 0.05) [18]. All plots were made using ggplot2 [19]. The division of X chromosome genes into classes was based on Tukiainen et al. [20].

#### Enrichment analysis

To investigate possible biological functions of the RNA expression changes in our cohort, we performed overrepresentation analysis (ORA) using Clusterprofiler [21]. Differentially expressed genes were used as input. Disease associations were identified by Disease Ontology (DO) enrichment analysis, while Gene Ontology Biological Processes (GOBP) were used for functional enrichment [22, 23]. The top-enriched terms for each enrichment analysis were sorted according to *p*-value and presented as barplots.

#### Weighted gene co-expression network analysis

To identify co-expressed genes from the RNA-seq data and relate these to the number of sex chromosomes, weighted correlation network analysis (WGCNA, v1.70.3) was applied [24]. For each tissue, outlier samples were detected using hierarchical sample clustering, removing one 47,XXY blood sample. A signed co-expression network was constructed for each tissue, using a one-step approach, calculating adjacency choosing an appropriate soft thresholding power with approximate scale-free topology. Gene clustering was performed on the signed Topology Overlap Matrix by hierarchical clustering, identifying modules via the blockwiseModules function with a minModuleSize of 30 and a mergeCutHeight of 0.25. The module eigengenes were calculated via the moduleEigengenes function, and eigengene significance and corresponding *p*-value were obtained for each module-trait association in each tissue. Intramodular connectivity and gene significance for the number of X or Y chromosomes were extracted for each module of interest. For modules of interest, hub genes were identified using the chooseTopHubInEachModule function. 

### Methylation analysis (Infinium MethylationEPIC)

#### Infinium MethylationEPIC

1 μg of genomic DNA from all three tissues was bisulfide converted using the Illumina iScan (platform). The methylation level was measured using the Infinium MethylationEPIC DNA Analysis BeadChip (Array type) at Eurofins Genomics AS. Shortly, the DNA was bisulfide converted and then analyzed using Illumina’s Infinium assay that relies on direct hybridization of genomic targets to array-bound sequences. Single base extension was followed by florescence staining, signal amplification, and scanning on an iScan instrument. The raw intensity values obtained from the Infinium MethylationEPIC chip was read into R (v. 4.1.1) and further processed using the R package Minfi [25]. We filtered out cross-reactive probes and poor performance probes with a detection *p*-value < 0.01. Next, we applied preprocessFunnorm normalization, a between-array normalization method that removes variation by regressing out variability inferred by the control probes [26]. Cross-reactive probes were then removed, and the methylation values were calculated as M-values (logit [beta]) (Equation (I)).

I $$M-value= \mathrm{log}2\left(\frac{Beta}{1-Beta}\right)$$

Multidimensional scaling plots, using the top 1000 CpG sites as input, were evaluated to identify clusters of samples behaving differently than expected. Finally, the probes were annotated to the human genome version38 (https://github.com/achilleasNP/IlluminaHumanMethylationEPICanno.ilm10b5.hg38.git). For differential methylation analysis, the M-values were analyzed using LIMMA (~ 0 + karyotype) [27]. Differentially methylated positions were defined as adj. *p*-value < 0.05 and delta M > 1.0. DMRcate [28] were used to identify DNA regions that were differentially methylated, using a false discovery rate = 0.05, adjusted *p*-value < 0.05 and a |mean M-value|> 0.1. We defined a DMR as a minimum of 2 consecutive CpGs. If there were more than 1000 nucleotides between significant CpG, the sites were divided into separate DMRs. DNA methylation has been shown to play an important role in modulating gene expression. Therefore, we correlated changes in methylation and gene expression. For the correlation analysis, all significant DMRs (adj. *P*-value < 0.05) were correlated with gene expression, also including non-significant expressed genes, in order to evaluate trends in methylation status and gene expression. These were plotted in a scatter plot based on log2FC and ∆M-value. In the second part of the analysis, we used Spearman’s rank correlation to analyze the correlation between the specific methylation level (M-value) and RNA expression level (normalized counts) for shared genes. We considered the correlations to be interesting (either negative or positive) if *p* < 0.1.

## Results

### X chromosome number affects X chromosomal transcription signature across tissue types

**Fig. 2:**
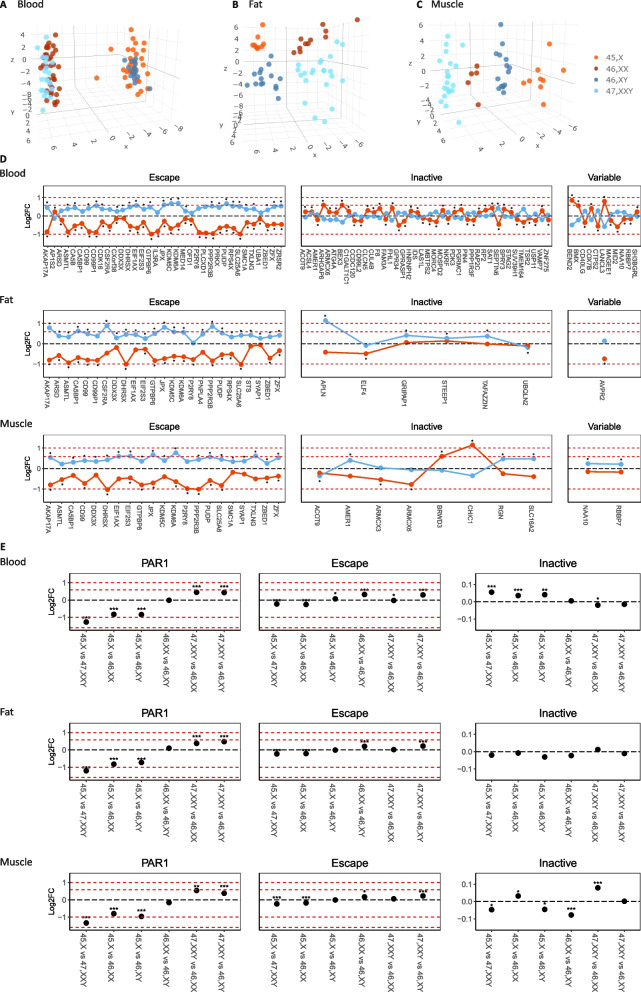
X chromosomal RNA expression. Unsupervised clustering of X chromosomal genes comparing 45,X (orangered), 46,XX (dark red), 46,XY (dark blue), and 47,XXY (light blue) in **A** blood, **B** fat, and **C** muscle. Dotplots demonstrating Log2 fold change of X chromosomal genes being differentially expressed between 45,X vs 46,XX or 47,XXY vs 46,XY or both and based on their X chromosome inactivation status (escape, inactive, variable) in blood, fat, and muscle (*p < 0.05) (**D**), and average group wise expression pattern of PAR1, escape and inactive genes on the X chromosome in blood, fat, and muscle (*p < 0.005, **p < 0.01, ***p < 0.001) (**E**). The red dashed lines visualize log 2FC at − 1, 0.58, and 1

First, we investigated if X chromosome number influenced X chromosomal gene expression in a tissue-specific way. Using unsupervised clustering, a clear clustering of 45,X, 46,XX, 46,XY, and 47,XXY was evident based on X chromosomal gene expression in fat and muscle (Fig. 2B, C), whereas in blood 45,X clustered with 46,XY and 47,XXY clustered with 46,XX (Fig. 2A). The observed pattern in fat and muscle indicated a possible effect of sex on X chromosomal gene expression. However, we cannot clearly separate the effect of sex from the presence of a Y chromosome, and the numbers of X chromosomes, as our cohort did not include samples from sex reversal conditions.

The impact of sex chromosome number on the X chromosomal expression pattern in blood (Additional file 3: Tables S1-S6) confirmed previous findings of predominantly gene downregulation in 45,X and gene upregulation in 47,XXY compared to controls with the same sex (Table 1) [14, 29, 30]. In fat and muscle (Additional file 3: Tables S7-S18), we observed the same pattern, both when comparing with same sex controls and opposite sex controls (Table 1).

**Table 1:** Up- and downregulated differentially expressed genes

		45,X vs 46,XX		45,X vs 46,XY		47,XXY vs 46,XX		47,XXY vs 46,XY	
		Up	Down	Up	Down	Up	Down	Up	Down
Blood	X chromosome	54	**91**	**60**	48	**15**	7	**40**	4
	Autosomes	**1409**	1244	**1763**	739	**216**	188	**149**	64
Fat	X chromosome	0	**30**	43	**60**	**85**	75	**38**	4
	Autosomes	47	**113**	**1885**	1656	2484	**2715**	**239**	118
Muscle	X chromosome	2	**18**	46	**67**	**50**	33	**29**	4
	Autosomes	20	**22**	**2148**	1770	1499	**1549**	**120**	74

Number of differentially expressed genes (adjusted *p*-value < 0.05, FDR < 0.05) both up- (log2FC > 0) and downregulated (log2FC < 0) in blood, fat, and muscle tissue in all four contrasts; 45,X vs. 46,XX, 45,X vs. 46,XY, 47,XXY vs. 46,XX, and 47,XXY vs. 46,XY. The bold font indicates if the majority of differentially expressed genes were either up- or downregulated in the given contrast

X chromosomal genes are classified into three categories according to their X inactivation status: (1) escape genes (genes that escape X inactivation), (2) inactive genes (genes inactivated by X inactivation), and (3) genes that show variable inactivation [20]. The escape genes also include the genes located in the pseudoautosomal region 1 (PAR1) of the X chromosome. Among differentially expressed X chromosomal genes (xDEGs), we found that escape genes and PAR1 genes were overrepresented in all three tissues, as expected, and that the majority of xDEGs were located on Xp (Additional file 1: Fig. S1).

We identified several overlapping xDEGs between 45,X vs. 46,XX and 47,XXY vs. 46,XY (Table 1). 14 xDEGs overlapped in all three tissues (Table 2, Additional file: Fig. S2). Of these, three (*JPX*, *TSIX*, and *XIST*) are known to be involved in X chromosome inactivation, seven (*AKAP17A*, *CD99*, *DHRSX*, *GTPBP6*, *PPP2R3B*, *SLC25A6*, *ZBED1*) are located in PAR1, and four (*EIF2S3*, *KDM6A*, *PUDP*, *ZFX*) are annotated as escape genes. Notably, 6 of these 11 genes have widespread regulatory genomic functions (Table 2).

**Table 2:** Overlapping differentially expressed X chromosomal genes in blood, fat and muscle

HGNC gene symbol	Location on X chromosome	XCI status	Function
JPX	XIC	Escape	Participate in X chromosome inactivation
TSIX	XIC	Variable escape	Participate in X chromosome inactivation
XIST	XIC	Mostly subjected to inactivation	Participate in X chromosome inactivation
AKAP17A	PAR 1 region	Escape	Regulatory genomic functions: Splicing
CD99	PAR 1 region	Escape	Involved in cell adhesion, migration, death, differentiation and diapedesis
DHRSX	PAR 1 region	Escape	Involved in regulation of autophagy
GTPBP6	PAR 1 region	Escape	Regulatory genomic functions: Translation
PPP2R3B	PAR 1 region	Escape	Involved in the regulation of cell growth and division
SLC25A6	PAR 1 region	Escape	Transporter activity and ATP:ADP antiporter activity
ZBED1	PAR 1 region	Escape	Regulatory genomic functions: Transcription
EIF2S3	Xp22.11	Escape	Regulatory genomic functions: Translation
KDM6A	Xp11.3	Escape	Regulatory genomic functions: Ubiquitination and chromatin modification
PUDP	Xp22.31	Escape	Unknown
ZFX	Xp22.11	Escape	Regulatory genomic functions: Transcription

Differentially expressed X chromosomal genes (adjusted *p*-value < 0.05 and FDR < 0.05) in all three tissues; blood, fat, and muscle. Table describes the location of the gene on the X chromosome, XCI (X chromosomal inactivation) status as escape, variable escape, inactive, and known function of the gene

Most genes annotated as escape genes were differentially expressed in 45,X vs. 46,XX (34 out of 46) and 47,XXY vs. 46,XY (26 out of 46) in blood (Fig. 2D). As expected, these genes were downregulated in 45,X vs. 46,XX and upregulated in 47,XXY vs. 46,XY, except *AP1S2* (Fig. 2D). The observed changes in gene expression agreed with previous studies showing that escape genes tend to be expressed from the inactive X chromosome (Xi) at a level that is between 10 and 95% the expression level seen from the active X chromosome (Xa) [31–33]. The same pattern was observed in fat and muscle tissue (Fig. 2D, Additional file 2: Text S1).

Of the genes annotated as inactive (blood, *n* = 254; fat, *n* = 315; muscle, *n* = 279), we identified 40 xDEGs in blood, six xDEGs in fat and eight xDEGs in muscle (differentially expressed in either 45,X vs. 46,XX, or 47,XXY vs. 46,XY, or both contrasts) (Fig. 2D). In blood, 18 of the 40 genes were downregulated and 22 upregulated in 45,X vs. 46,XX (Fig. 2D) with eight of these genes (*ACSL4*, *ARHGAP6*, *ARMCX6*, *CLCN5*, *MOSPD2*, *RP2*, *SEPTIN6*, *ZNF275*) showing the same expression pattern as demonstrated by Zhang et al. [34]. Only one gene, *SEPTIN6*, was found to be differentially expressed between 47,XXY vs. 46,XY in blood (Fig. 2D) [34].

To further examine the expression profile of X chromosomal genes in relation to X and Y chromosome number, we analyzed the change in expression level of PAR1 genes, escape genes, and inactive genes in the four karyotype groups (Fig. 2E, Additional file 1: Fig. S3). The mean change in expression of PAR1 genes between the karyotype groups in all three tissue types were as we would expect based on sex chromosome number and previous studies demonstrating a minor spread of inactivation into the PAR1 region of Xi in individuals with two X chromosomes, leading to higher expression of PAR1 genes among males compared with females [31]. Also, the mean changes in expression of escape genes between karyotype groups in all three tissues were as we would expect based on X chromosome number and evidence that escape genes are not fully expressed form the Xi [31, 32]. A tissue-specific-expression pattern was seen for inactive genes (Fig. 2E). In blood, the mean expression level of inactive genes in groups with more than one X chromosome (e.g., 46,XX and 47,XXY) were less than the mean expression in 45,X, indicating that the expression of genes on the Xa is to some extent also repressed. Notably, we found that the gene expression changes in 45,X vs. 46,XX and 45,X vs. 46,XY had the same level (Fig. 2E), indicating that Y chromosomal genes may affect the expression of inactive genes on the active X chromosome (Xa). Furthermore, the log2FC of 45,X vs. 47,XXY was higher than both 45,X vs. 46,XX and 45,X vs. 46,XY (Fig. 2E), indicating that as the number of sex chromosome increases, the expression of genes annotated as inactive on the Xa decreases—perhaps as a compensatory mechanism. In muscle tissue, we observed the same pattern as in blood for 45,X vs. 46,XX. However, when making comparisons where one of the groups had a Y chromosome (e.g., 46,XY or 47,XXY), we observed that the overall expression of inactive genes was increased in groups with only X chromosome(s) (e.g., 45,X or 46,XY), indicating that in muscle, Y chromosomal genes may impact the expression of inactive genes. In fat tissue, we observed the expected pattern of inactive genes, with no significant differences in mean expression between groups with different X chromosome numbers.

### The effect of X chromosome number is genome-wide

**Fig. 3:**
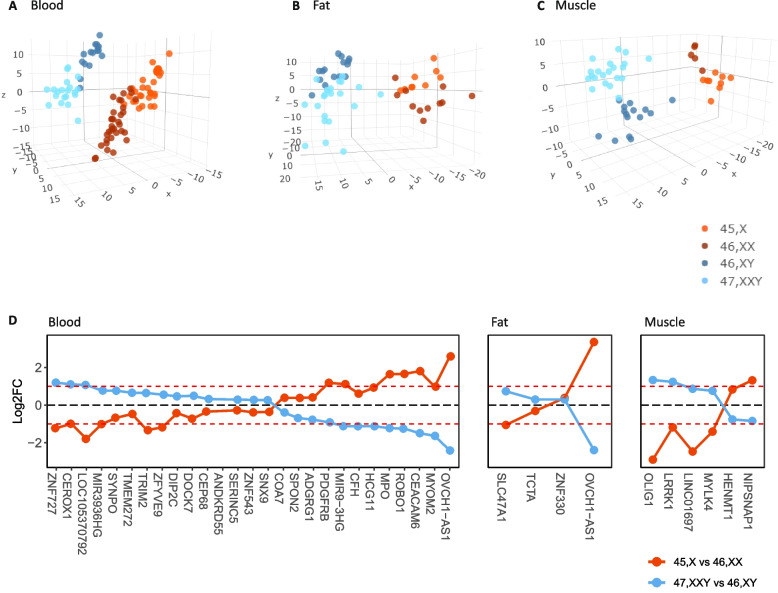
Autosomal RNA expression. Unsupervised clustering of X chromosomal genes comparing 45,X (orange red), 46,XX (dark red), 46,XY (dark blue), and 47,XXY (light blue) in **A** blood, **B** fat, and **C** muscle. Dotplots demonstrating Log2 fold change autosomal genes being differentially expressed between both 45,X vs 46,XX and 47,XXY vs 47,XY in blood, fat, and muscle (**D**). The red dashed lines visualize log 2FC at − 1 and 1

Previous studies have documented a genome-wide effect of X chromosome number in blood [14, 29, 34, 35]. Our differential expression analysis of autosomal genes revealed the same genome-wide effect in blood as well as in fat and muscle (Table 1, Additional file 1: Figs. S4 and S5, Additional file 3: Tables S19-S36). Unsupervised clustering revealed a clear genotype-specific clustering in all three tissues based on autosomal genes (Fig. 3A–C). Four autosomal differentially expressed genes (aDEGs) in fat, 6 aDEGs in muscle, and 27 aDEGs in blood were shared between 45X vs. 46,XX and 47,XXY vs. 46,XY (Fig. 3D). Notably, all 37 aDEGs, except one, displayed an inverse expression pattern in 45,X vs. 46,XX compared to 47,XXY vs. 46,XY, being either upregulated in 45,X vs. 46,XX and downregulated in 47,XXY vs. 46,XY or vice versa (Fig. 3D). Only few aDEGs in 45,X vs. 46,XX overlapped across all three tissues (*CCDC28A*,* OVCH1-AS1*) and in 47,XXY vs. 46,XY only one overlapping aDEG was found (*DDX43*) (Fig. 3D, Additional file 1: Fig. S6).

### Differentially expressed genes are associated with comorbidities in 45,X and 47,XXY and reveal distinct co-expression patterns

**Fig. 4:**
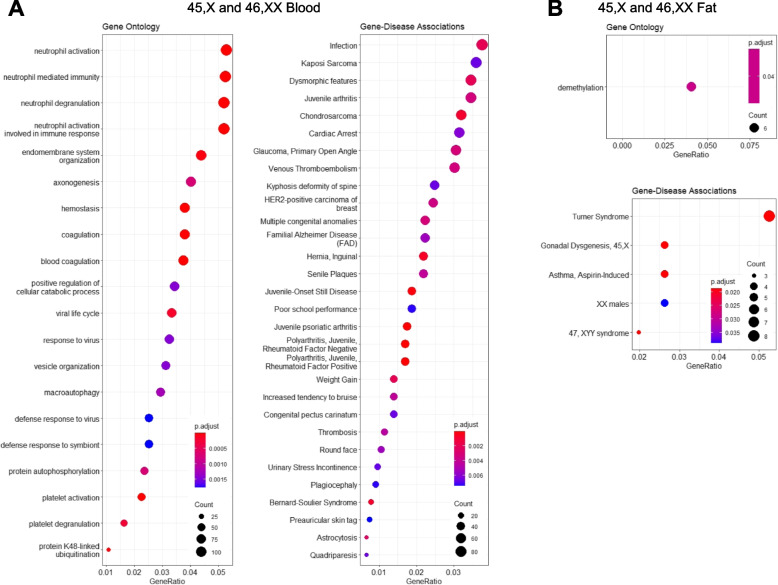

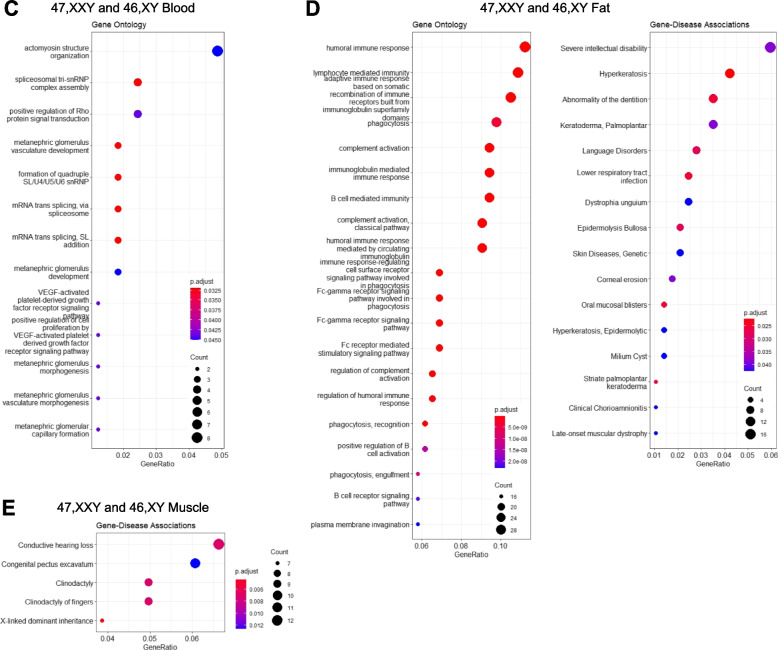
Enrichment analysis. Enrichment analysis of differentially expressed genes between 45,X and 46,XX in blood (**A**) and fat (**B**). Enrichment analysis of differentially expressed genes between 47,XXY and 46,XY in blood (**C**), fat (**D**), and muscle (**E**)

To gain insight into the biological function of the changes seen in the transcriptome of 45,X and 47,XXY, we performed enrichment analysis using all DEGs as input (padj < 0.05) (aDEGs and xDEGs). Enrichment was found for several terms related to known comorbidities in 45,X and 47,XXY (immune system, inflammatory diseases, skin diseases, neurodegenerative disorders, language disorders, abnormalities of dentition, hearing loss, congenital malformations, coagulation) with few overlaps between tissue types (Fig. 4, Additional file 2: Text S2).

To further study the transcriptional impact of SCAs, weighted gene correlation network analysis (WGCNA) was applied to identify co-expressed groups of genes (Additional file 1: Figs. S7-S10). Gene expression data from each tissue was used as input, firstly from all chromosomes and secondly restricted to the autosomes. We looked for strong correlations between each module of co-expressed genes and (1) karyotype and (2) the number of sex chromosomes (Additional file 1: Figs. S7 and S9). Here, the number of Y chromosomes represented sex (46,XY and 47,XXY vs. 45,X and 46,XX), whereas the number of X chromosomes represented the X number, independent of sex (46,XX and 47,XXY vs. 45,X and 46,XY). Within strongly correlated modules (highlighted in Additional file 1: Fig. S7), we further investigated the correlation between each gene and trait (Additional file 1: Fig. S8).

When including genes from both autosomes and sex chromosomes, one module from each tissue type was strongly associated with the number of X chromosomes (“royalblue” for blood, “skyblue3” for fat and “darkolivegreen” for muscle) (Additional file 1: Fig. S7A, B, C), indicating that the expression of these sets of genes were dependent on X chromosome number. As expected, these modules contained many X-linked genes, primarily from PAR1 or with Y-linked homologs. Common genes for the three tissue modules were *XIST*, *TSIX*, *AKAP17A*, *KDM6A*, *PUDP*, *KDM5C*, and *ZFX* (Additional file 1: Fig. S8). One module in blood was correlated to the number of Y chromosomes (“darkorange” module) (Additional file 1: Fig. S7A), whereas several modules in fat and muscle were both positively and negatively associated with the number of Y chromosomes (Additional file 1: Fig. S7B, C). Again, this showed a stronger and more complex influence of sex on gene expression in fat and muscle compared to blood. As expected, the modules that were positively correlated to the number of X or Y chromosomes were positively correlated to 47,XXY and negatively correlated to 45,X (Additional file 1: Fig. S7), in line with the sex chromosome number.

We then identified expression patterns solely from autosomes to investigate potential ripple effects of SCAs on autosomal gene expression. A single module was associated with the number of X chromosomes in blood and muscle (“greenyellow” for blood, “lightgreen” for muscle) (Additional file 1: Figs. S9A, C and S10). However, these correlations were less significant and no convincing associations to the X count were detected in fat (Additional file 1: Fig. S9B). Again, several modules in fat and muscle were associated with the presence of a Y chromosome (Additional file 1: Fig. S9B, C), showing that the sex influence on fat and muscle is stronger than in blood when only considering autosomal genes.

### The impact of X chromosome number and sex on DNA methylation across tissue types

**Fig. 5:**
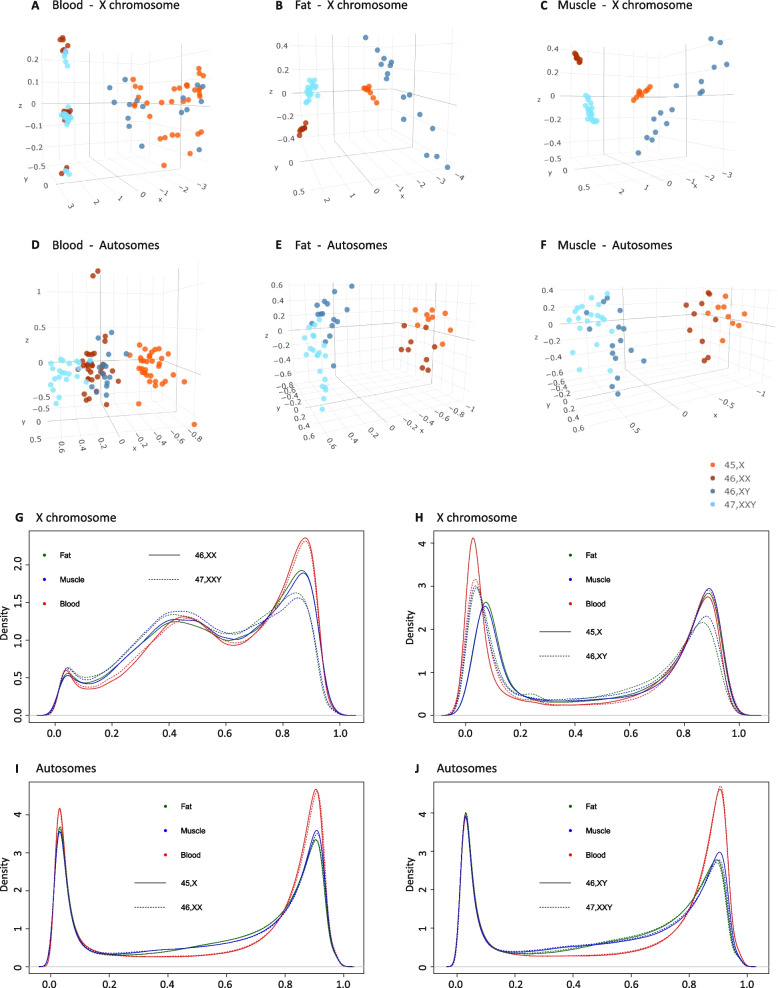
X chromosomal and autosomal DNA methylation. Multidimensional scaling of X chromosomal methylation sites in **A** blood, **B** fat, and **C** muscle. Multidimensional scaling of autsosomal methylation sites in **D** blood, **E** fat, and **F** muscle. Density plots of the distribution of all X chromosomal (**G**, **H**) and all autosomal (**I**, **J**) CpG sites methylation values (beta values) in blood (red), fat (green), and muscle (blue) in 45,X, 46,XX, 46,XY, and 47,XXY. **G** Distribution of beta values of X chromosomal CpG sites in 46,XX and 47,XXY. **H** Distribution of beta values of X chromosomal CpG sites in 45,X and 46,XY. **I** Distribution of beta values of autosomal CpG sites in 45,X and 46,XX. **J** Distribution of beta values of autosomal CpG sites in 46,XY and 47,XXY

To investigate the impact of X chromosome number and sex on the DNA methylation profile, we performed unsupervised clustering based on all X chromosomal and autosomal CpG methylation sites. In blood, based on X chromosomal methylation sites in blood, a distinct clustering based on X chromosome number was revealed. 45,X and 46,XY clustered together and 46,XX and 47,XXY clustered together (Fig. 5A*)*. In fat and muscle, a possible effect of sex and X chromosome number was observed (Fig. 5B, C). Based on autosomal methylation sites, a 45,X cluster and a 47,XXY cluster were observed, whereas 46,XX and 46,XY clustered closely together (Fig. 5D), in agreement with previous studies [14, 29]. When extending our analysis to fat and muscle tissue, we also observed a possible effect of sex and X chromosome number (Fig. 5E, F).

To further characterize these tissue differences, we investigated the distribution of methylation values in the four karyotype groups across tissues. On the X chromosome, 45,X had a higher density of hypomethylated sites and a lower density of hypermethylated sites in blood compared to 46,XY (Fig. 5G). To our surprise, the opposite was true for the distribution of methylation values in fat and muscle tissue. Here, we observed a lower density of hypomethylated and higher density of hypermethylated sites in 45,X compared to 46,XY (Fig. 5H). When comparing the distribution of methylation values on the X chromosome between 47,XXY and 46,XX, the same density of hypomethylated and hypermethylated sites were seen in blood (Fig. 5H). In muscle and fat, 47,XXY had a lower density of hypermethylated sites and a higher density of sites with an intermediary methylation level compared to 46,XX (Fig. 5H). On autosomes, the distribution of methylation values in all three tissues were similar for 45,X and 46,XX, showing a normal biphasic distribution (Fig. 5I). The same pattern was observed for 47,XXY and 46,XY, but with a tendency for 47,XXY to have a higher density of hypermethylated sites in muscle (Fig. 5J). These data illustrate that the impact of X chromosome number on the methylome is highly tissue-specific and differentially affected by sex.

The number of differentially methylated positions (DMPs) revealed a genome-wide effect of altered X chromosome number on methylation, across all three tissues (Table 3, Additional file 1: Fig. S11). Comparing 45,X vs. 46,XX, the majority of DMPs were hypomethylated across all tissues, while comparing 47,XXY vs. 46,XY the majority of DMPs were hypermethylated (Table 3), consistent with previous findings of an inverse methylation profile from peripheral blood [34]. The few autosomal DMPs (aDMPs) that overlapped between tissues were hypomethylated in 45,X and hypermethylated in 47,XXY, while the overlapping X chromosomal DMPs (xDMPs) were hypermethylated in 45,X and hypomethylated in 47,XXY (Additional file 1: Fig. S12). This inverse methylation pattern reflected the number of sex chromosomes as the comparison used both 46,XX and 46,XY as controls, thereby minimizing the effect of sex.

**Table 3:** Hyper- and hypomethylated positions

		45,X vs 46,XX		45,X vs 46,XY		47,XXY vs 46,XX		47,XXY vs 46,XY	
		Hyper	Hypo	Hyper	Hypo	Hyper	Hypo	Hyper	Hypo
Blood	X chromosome	2160	**6798**	4	**94**	1	**4**	**5825**	2924
	Autosomes	228	**820**	222	**696**	**151**	42	**287**	71
Fat	X chromosome	3063	**4502**	**1692**	2	4	**12**	**5207**	2261
	Autosomes	70	**201**	**1073**	160	101	**552**	**126**	49
Muscle	X chromosome	3521	**4389**	**2698**	2	2	**17**	**5041**	2773
	Autosomes	**316**	92	**6909**	132	102	**4256**	54	**88**

Number of differentially hyper- and hypomethylated positions in blood, fat, and muscle tissue, across all four contrasts; 45,X vs. 46,XX, 45,X vs. 46,XY, 47,XXY vs. 46,XX, and 47,XXY vs. 46,XY. The bold font indicates if the majority of positions were either hyper- or hypomethylated in the given contrast

### Analysis of DMPs in relation to genic location adds further evidence of an inverse methylation pattern between 45,X and 47,XXY

It is well established that CpG islands are usually methylated on the inactive X genes, while gene bodies are unmethylated. Furthermore, hypermethylation of X chromosomal gene bodies are correlated with gene expression [36–38]. We analyzed the identified DMPs in relation to genic locations. The DMPs were annotated according to their relation to gene, using eight annotation categories (1st Exon, 3′UTR, 5′ UTR, Body, Exon boundaries, TSS1500, TSS200, Unknown). The genic location of hypermethylated xDMPs in 45,X vs. 46,XY and hypomethylated xDMPs in 47,XXY vs. 46,XX were primarily localized in gene bodies in all three tissues (Additional file 1: Fig. S13A, B, C). Hypomethylated xDMPs in 45,X vs. 46,XY were primarily located at 5′UTR, TSS200, and TSS1500 in fat and muscle, but primarily in gene body in blood (Additional file 1: Fig. S13A, B, C).

Hypermethylated xDMPs in 47,XXY vs. 46,XX were also localized at 5′UTR and TS200 in fat, muscle, and blood. The distribution of aDMPs in relation to genic location was similar across all three tissues (Additional file 1: Fig. S13D, E). In conclusion, we demonstrate a methylation landscape in relation to genic location that presents as a mirrored image between 45,X and 47,XXY suggesting a common mechanism affecting methylation at specific CpG sites in both 45,X and 47,XXY. Furthermore, the methylation changes observed in muscle and fat may have larger functional effect because of their location within 5′UTR, TSS200, and TSS1500.

### 45,X and 47,XXY impacts the landscape of differentially methylated regions

Since changes in regional methylation have been shown to play a functional role in gene regulation [39], we investigated if 45,X and 47,XXY had any contrasting differentially methylated regions (DMRs) (Table 4).

**Table 4:** Differentially methylated regions

		45,X vs 46,XX	45,X vs 46,XY	47,XXY vs 46,XX	47,XXY vs 46,XY
Blood	X chromosome	1028	22	0	1013
	Autosomes	106	98	15	48
Fat	X chromosome	909	226	1	930
	Autosomes	29	275	349	16
Muscle	X chromosome	957	180	5	959
	Autosomes	86	2166	1526	19

Number of differentially methylated regions, defined as mean absolute M-value > 0.1, FDR < 0.05, adjusted *p*-value < 0.05, and number of CpG > 1, in blood, fat, and muscle tissue across all four contrasts; 45,X vs. 46,XX, 45,X vs. 46,XY, 47,XXY vs. 46,XX, and 47,XXY vs. 46,XY

Groups with unequal number of X chromosomes (47,XXY vs. 46,XY and 45,X vs. 46,XX) exhibited the largest difference in X chromosomal DMRs, while 47,XXY vs. 46,XX and 45,X vs. 46,XY had similar numbers of DMRs (Table 4). The number of autosomal DMRs in the same sex comparisons 47,XXY vs. 46,XY and 45,X vs. 46,XX were similar across all tissues (Table 4). This indicates that both X chromosome number and sex may impact regional methylation—with a more pronounced effect on methylation when lacking an X chromosome (45,X) compared with an additional X chromosome (47,XXY) (Tables 3 and 4).

### DMPs with methylation patterns in opposite directions in 45,X and 47,XXY have a stronger impact on autosomal gene expression in 45,X

Epigenetic mechanisms including DNA methylation can regulate gene expression, mediating gene silencing, or activation [40]. To investigate if the inverse DNA methylation pattern between 45,X and 47,XXY was reflected in the transcriptome, we correlated aDMPs and xDMPs in both 45,X and 47,XXY to gene expression (adjp < 0.05, absolute delta M-value > 0.3) (Additional file 1: Fig. S14, Additional file 2: Text S3). Autosomal DNA methylation affected autosomal gene expression more severely in 45,X compared to 47,XXY, as a larger proportion of the autosomal genes annotated to the aDMPs were differentially expressed in 45,X vs. 46,XX compared with the 47,XXY vs. 46,XY contrast (Additional file 1: Fig. S14).

In blood, three autosomal genes (*OR2C3*,* TRIM2*, and *HCG11*) and one X chromosomal gene (*KDM5C*) were hypermethylated in 47,XXY and hypomethylated in 45,X and also differentially expressed in both 45,X vs 46,XX and 47,XXY vs 46,XY. Six autosomal genes *(NOVA1*,* ANKRD55*,* DIP2C*,* SORBS2*,* SERINC5*,* SRGAP1*) and one X chromosomal gene (*ARSD*) were differentially expressed and hypomethylated in 47,XXY and hypermethylated in 45,X (Additional file 1: Fig. S14). In muscle, only one autosomal gene, *HENMT1*, hypermethylated in 47,XXY and hypomethylated in 45,X, and one X chromosomal gene, *EIF2S3*, hypomethylated in 47,XXY and hypermethylated in 45,X was observed (Additional file 1: Fig. S14).

### Correlation between DMRs and DEGs across tissues identified more X chromosomal DMR/DEGs than autosomal

We defined a DMR/DEG pair as a gene that was differentially expressed (adjp < 0.05) and associated with a significant differentially methylated region annotation (*p*-value < 0.05, absolute mean M-value > 0.1 and CpG > 1). Overall, the correlation between DMRs and DEGs was sparse across all comparisons (Table 5, Additional file 1: Fig. S15, Additional file 2: Text S4). However, one gene was of particular interest, *KDM6A*, identified as a xDMR/xDEG in both 45,X vs. 46,XX and 47,XXY vs. 46,XY in both blood, fat, and muscle. This DMR/DEG pair was hypomethylated and upregulated in 47,XXY, while hypermethylated and downregulated in 45,X.

**Table 5:** Number of differentially expressed genes related to a differentially methylated region

		45,X vs 46,XX	45,X vs 46,XY	47,XXY vs 46,XX	47,XXY vs 46,XY
Blood	X chromosome	88	2	0	20
	Autosomes	9	8	1	2
Fat	X chromosome	2	9	0	11
	Autosomes	0	21	40	0
Muscle	X chromosome	4	17	0	18
	Autosomes	1	163	97	0

Number of differentially expressed genes related to a differentially methylated region in blood, fat, and muscle across all four contrasts; 45,X vs. 46,XX, 45,X vs. 46,XY, 47,XXY vs. 46,XX, and 47,XXY vs. 46,XY

## Discussion

In this study, we present a comprehensive and integrative analysis of the transcriptome and methylome in three different phenotypically relevant tissues, across four karyotype groups; 45,X, 46,XX, 46,XY, and 47,XXY. To our knowledge, this is the first study analyzing the impact of X chromosome number on the transcriptome and methylome across multiple tissues from the same individuals. Our results significantly add to the emerging literature of the genetic and epigenetic architecture of SCAs.

Collectively, we demonstrate that the inverse genome-wide transcriptome profile in 45,X and 47,XXY, previously described in blood, can be extended to other tissue types of the same embryonic origin (e.g., mesoderm). However, our data also revealed that the effect of X chromosome number is tissue-specific and further indicate a possible effect of sex and presence or absence of the Y chromosome on the transcriptome in fat and muscle.

The higher number of DEGs between 45,X and 46,XX in blood is partly reflected by the larger number of blood samples. Using random down-sizing of the number of blood samples, before differential expression analysis, to reflect the number of fat and muscle samples resulted in 469 DEGs and 412 DEGs, respectively.

The similar expression patterns in the different tissue types indicate the existence of a universal X chromosome number dependent gene-regulatory mechanism in SCAs. Such a gene-regulatory mechanism may be initiated by escape genes having transcriptional regulatory functions and being able to start a cascade of regulatory pathways which in the end lead to the expression pattern illustrated. Evidence of a common gene-regulatory mechanism in SCAs comes from the finding of overlapping xDEGs between 45,X and 47,XXY across tissues displaying an inverse expression pattern. These genes—*AKAP17A*, *CD99*, *DHRSX*, *EIF2S3*, *GTPBP6*, *JPX*, *KDM6A*, *PP2R3B*, *PUDP*, *SLC25A6*, *TSIX*, *XIST*, *ZBED1*, *ZFX*—may be central in the epigenetic regulation of sex chromosome aneuploidies. Zhang et al. demonstrated that *ZFX* binding motifs were significantly enriched in autosomal promoter and enhancer regions of aDEGs in 45,X, but not in 47,XXY [34]. *ZFX*, encoding a transcriptional factor, has previously been proven differentially expressed in blood from 45,X and 47,XXY [14, 29, 34]. Thus, *ZFX* could be a central gene initiating the ripple effect of SCAs on the genome. Moreover, we also identified several shared aDEGs between 45,X and 47,XXY in blood, fat, and muscle, respectively. All, except one of these, displayed an inverse expression profile, further indicating a shared genomic regulatory mechanism of SCAs. The interplay identified between X chromosomes and autosomes was supported by the WGCNA analysis, showing that gene networks from the autosomes could be associated to with the number of X chromosome and that altered X chromosome number drives more subtle, tissue-dependent, changes to a larger set of autosomal genes.

As anticipated, most PAR1 genes, across all three tissues, were downregulated in 45,X, while upregulated in 47,XXY. This agrees with previous studies performed on blood cells [34, 35]. In addition, we also demonstrated that PAR1 genes display an expression difference between 45,X vs. 46,XX and 47,XXY vs. 46,XY that followed the expected pattern based on sex chromosome number and previous studies demonstrating a minor spread of inactivation into the PAR1 region of Xi [31]. Notably, in muscle, *SHOX* was upregulated in 45,X vs. 47,XXY, and a trend towards upregulation in 45,X vs. 46,XX and downregulation in 47,XXY vs. 46,XY was observed. *SHOX* is involved in growth and expression changes in SHOX drive differences in height [41, 42]. *SHOX* is normally expressed in skeletal muscle and is typically downregulated in females compared with males [20]. Since counts in all 4 karyotypes were low (< 200), this finding should be interpreted with caution, but could suggest that some PAR1 genes can exhibit tissue-specific expression pattern. However, it is also possible that temporal variation in SHOX expression may exist and that high levels of *SHOX* is only expressed in temporal windows during embryonic development and during rapid growth in childhood [43, 44].

In addition, we showed that some escape genes are not differentially expressed between 45,X vs. 46,XX and 47,XXY vs. 46,XY. This could indicate that regulatory mechanisms are at play in 45,X and 47,XXY to diminish the gene imbalance of an altered X chromosome number. Moreover, we showed that some escape genes and inactive genes were differentially expressed in 45,X but not in 47,XXY, or vice versa, indicating that some regulatory mechanism may be specific for the specific SCA subtypes. Previous findings on peripheral blood showed a similar expression pattern in 24 out of 28 escape genes in 45,X vs. 46,XX and of 27 out of 32 escape genes in 47,XXY vs. 46,XY [34]. Our data is also in agreement with a study performed on lymphoblastoid cell lines, demonstrating that some escape genes show a sublinear increase in expression in relation to increased X chromosome number in blood [35].

Based on our analyses, some genes annotated as inactivated were differentially expressed between 45,X and 46,XX, 47,XXY and 46,X or both, adding them to the pool of possible X chromosomal candidate genes involved in the phenotypic traits of 45,X and 47,XXY. Interestingly, our results indicate that not only genes annotated as inactive are inactivated on the Xi, but that these genes are also repressed on the Xa in individuals with more than one X chromosome, as previously reported [35].

In addition, we also found evidence that Y chromosomal genes may have the ability to repress the expression of genes on Xa and increase expression of genes from the Xi. However, these findings need further validation, before any conclusions can be drawn.

Collectively, our findings elucidate a much more complex relationship between X chromosomal gene expression and X chromosome number. These alterations may be caused by altered expression level of transcription factors and could be a compensatory mechanism to dampen the genomic impact of altered X chromosome number. However, these changes could also play an important role in the phenotype of SCAs.

Our analysis also revealed a tissue-specific and genome-wide impact of X chromosome number on the methylome. 45,X and 47,XXY demonstrated a unique methylation profile in all three tissues. Furthermore, our analysis indicated a possible impact of sex on the methylome in fat and muscle. We demonstrated that the general hypomethylation in 45,X and hypermethylation in 47,XXY in blood, described previously [14, 29, 34], could not be extended to fat and muscle tissue. In muscle tissue, 45,X was associated with autosomal hypermethylation compared to both 46,XX and 46,XY, while 47,XXY was associated with autosomal hypomethylation compared to both 46,XX and 46,XY. This pattern of autosomal methylation in 45,X and 47,XXY in muscle, is in agreement with the finding that 46,XY males present with hypomethylation of autosomal genes involved in muscle contraction and substrate metabolism compared with 46,XX females [45]. However, 47,XXY were associated with autosomal hypomethylation and 45,X with autosomal hypermethylated when compared to controls of the same sex. These altered autosomal methylation patterns in muscle compared to controls of the same sex may be linked to the increased risk of the metabolic syndrome and type 2 diabetes frequently seen in 45,X and 47,XXY [3, 46, 47].

Our finding that loss of an X chromosome had a more severe impact on DNA methylation compared with an additional X chromosome is in line with a previous study [30]. This may reflect a comparatively effective X inactivation of the additional X in 47,XXY and/or the lack of two copies of PAR genes in 45,X necessary for obtaining epigenetic balance [48]. Nevertheless, it is evident that common epigenetic regulatory mechanisms are at play in 45,X and 47,XXY, as 45,X and 47,XXY share many DMPs that display an inverse methylation pattern. This pattern has been demonstrated for blood [30, 34], but here we also show the existence of such a pattern in fat and muscle.

Previous studies have documented changes in the methylome and transcriptome in 45,X and 47,XXY to be complementary rather than overlapping [14, 34]. Here, we demonstrate several X chromosomal and autosomal genes that are both differentially methylated at single CpG sites and differentially expressed. We also demonstrate several X chromosomal and autosomal DMR/DEGs across all three tissues, although mostly prevalent in blood and mostly on the X chromosome. Some of these genes could warrant further attention, as they may be related to the phenotype of SCAs. *CACNA2D1* is of interest as it was found to be hypomethylated and upregulated in 45,X in fat tissue. *CACNA2D1* encodes a protein in the voltage-dependent calcium channel complex, which is essential in neurotransmission, and is associated with epilepsy [49], intellectual disability [50], and cardiac arrhythmias [51] which are conditions with an increased prevalence in 45,X [3, 12, 52]. *STS* and *APLN*, both hypomethylated and upregulated in 47,XXY in fat tissue, also seem to be of special interest. *STS* belongs to the sulfatase family that catalyzes the conversion of estrogen, cholesterol, and androgen precursors into their active form [53]. It is associated with increased blood pressure [54], which is a common comorbidity in sex chromosome abnormalities [46, 55]. *APLN* encodes a peptide hormone secreted by adipocytes, involved in the regulation of various metabolic functions and upregulated by insulin and obesity [56]. *APLN* expression has been positively correlated with total cholesterol and LDL cholesterol [57]. In muscle tissue, *PWWP2B* and *THPO* could be of special interest*. PWWP2B* was hypermethylated and downregulated in 45,X. Lack of *PWWP2B* promotes adipocyte thermogenesis [58]. *THPO* was hypermethylated and downregulated in 45,X. *THPO* encodes a cytokine that regulates platelet production and proliferation and controls thrombopoiesis [59]. Dysregulation of *THPO* has been implicated in cardiovascular diseases, which are prevalent in 45,X and 47,XXY [11, 60].

Of the observed DMR/DEG pairs, *PRRG1* was of particular interest. *PRRG1* encodes a vitamin K-dependent transmembrane protein and diseases associated with *PRRG1* are involved in coagulation defects. Furthermore, a decrease in *PRRG1* (as seen in 47,XXY) is associated with intima thickening in atherosclerosis [61]. *SLC16A*2 was hypomethylated and upregulated in blood in 45,X while hypermethylated and downregulated in 47,XXY in both blood and muscle tissue. *SLC16A2* has been proposed involved in development of the central nervous system, while loss of function mutations has been associated with psychomotor retardation in males while females exhibit no neurological defects [62]. Mutations in *SLC16A2* are the cause of Allan-Herndon-Dudley syndrome, associated with impaired thyroid metabolism, presenting with neurodevelopmental delay, hypertonia, and muscle weakness [63]. Both 47,XXY and 45,X suffer from thyroid disorders, and especially in 47,XXY neurocognition is affected, correlating well with downregulation of this gene [6]. *MID2* was hypomethylated and upregulated in 45,X in both blood and muscle tissue. *MID2* is associated with Opitz syndrome, a primary midline development disease characterized by congenital facial malformation, abnormalities of the central nervous system (including motor skill defects and developmental delay), and congenital heart defects [64, 65]. This gene was only affected in 45,X and could perhaps be involved in cardiac septal defects and coarctation of the aorta in 45,X [66].

*KDM6A*, has previously been found differentially expressed and methylated in blood from 45,X [14, 34]. Here, we demonstrate that *KDM6A* is a DMR/DEG pair in both 45,X and 47,XXY across all three tissues. *KDM6A* is thought to affect cardiac looping, neural tube development [67], germ cell development [68], and neurocognition and global growth and development of seizures [69]. *MCF2* was hypermethylated and upregulated in 47,XXY and hypomethylated in 45,X across all three tissues. Differential methylation related to *MCF2* is associated with several psychiatric disorders [70].

In muscle, one aDMR/DEG was of particular interest; *PRKAG2*, which was both hypermethylated and downregulated in 45,X. *PRKAG2* encodes a subunit of the AMP activated protein kinase (AMPK) regulating muscle glucose metabolism and glycogen storage [71]. In response to reduced intracellular ATP levels, AMPK activates energy-producing pathways and inhibits biosynthesis of protein, carbohydrate, and lipids [72]. It is expressed in both skeletal muscle and cardiac muscle and has been shown to be involved in cardiac conduction disorders [73]. Furthermore, dysfunctional mutations in *PRKAG2* have been proposed in liver cirrhosis [74]. Both liver disease and cardiac arrhythmias are prevalent in 45,X [3, 60].

## Conclusion

The present study on the underlying genomics of 45,X and 47,XXY syndromes extends previous studies done on blood to other phenotypically relevant tissues and shows that there are both global and tissue-specific changes in both the methylome and transcriptome. We describe an inverse pattern of methylation and gene expression between 45,X and 47,XXY, a pattern most pronounced for X chromosomal genes. Furthermore, we point towards several new candidate genes. Our results add significantly to the emerging literature stating evidence of a comprehensive and complex X chromosome number effect on the human genome [34, 35].

## Supplementary Information

**Additional file 1: Figure S1.** Manhattan plots of X chromosomal differentially expressed genes. **Figure S2**. Venn diagrams of X chromosomal differentially expressed genes. **Figure S3.** Circular plots of PAR genes. **Figure S4.** Manhattan plots of autosomal differentially expressed genes. **Figure S5.** Densityplot of autosomal differentially expressed genes (aDEGs). **Figure S6.** Venn diagrams of autosomal differentially expressed genes. **Figure S7.** WGCNA analysis module 1–3. **Figure S8.** WGCNA analysis trait 1–3. **Figure S9.** WGCNA analysis module 4–6. **Figure S10.** WGCNA analysis trait 4–5. **Figure S11.** Venn diagrams of X chromosomal and autosomal differentially methylated sites. **Figure S12.** Venn diagram of X chromosomal and autosomal differentially methylated sites. **Figure S13.** Distribution of X chromosomal differentially methylated sites (xDMPs) and autosomal differentially methylated sites (aDMPs) between different contrasts in relation to genic region. **Figure S14.** Correlation plots of significant differentially methylated positions (DMPs) in correlation with gene expression. **Figure S15.** Correlation plots of significantly differentially methylated regions (DMRs) in correlation with gene expression.

**Additional file 2: Text S1.** Expression profile of escape genes in fat and muscle. **Text S2.** Enrichment analysis of DEGs. **Text S3.** Correlation between DMPs and gene expression. **Text S4.** Correlation between DMRs and DEGs across tissues.

**Additional file 3: Table S1.** X chromosomal DEGs between 45,X vs 46,XX in blood. **Table S2.** X chromosomal DEGs between 45,X vs 47,XXY in blood. **Table S3.** X chromosomal DEGs between 45,X vs 46,XY in blood. **Table S4.** X chromosomal DEGs between 46,XX vs 46,XY in blood. **Table S5.** X chromosomal DEGs between 47,XXY vs 46,XX in blood. **Table S6.** X chromosomal DEGs between 47,XXY vs 46,XY in blood. **Table S7.** X chromosomal DEGs between 45,X vs 46,XX in fat. **Table S8.** X chromosomal DEGs between 45,X vs 47,XXY in fat. **Table S9.** X chromosomal DEGs between 45,X vs 46,XY in fat. **Table S10.** X chromosomal DEGs between 46,XX vs 46,XY in fat. **Table S11.** X chromosomal DEGs between 47,XXY vs 46,XX in fat. **Table S12.** X chromosomal DEGs between 47,XXY vs 46,XY in fat. **Table S13.** X chromosomal DEGs between 45,X vs 46,XX in muscle. **Table S14.** X chromosomal DEGs between 45,X vs 47,XXY in muscle. **Table S15.** X chromosomal DEGs between 45,X vs 46,XY in muscle. **Table S16.** X chromosomal DEGs between 46,XX vs 46,XY in muscle. **Table S17.** X chromosomal DEGs between 47,XXY vs 46,XX in muscle. **Table S18.** X chromosomal DEGs between 47,XXY vs 46,XY in muscle. **Table S19.** Autosomal DEGs between 45,X vs 46,XX in blood. **Table S20.** Autosomal DEGs between 45,X vs 47,XXY in blood. **Table S21.** Autosomal DEGs between 45,X vs 46,XY in blood. **Table S22.** Autosomal DEGs between 46,XX vs 46,XY in blood. **Table S23.** Autosomal DEGs between 47,XXY vs 46,XX in blood. **Table S24.** Autosomal DEGs between 47,XXY vs 46,XY in blood. **Table S25.** Autosomal DEGs between 45,X vs 46,XX in fat. **Table S26.** Autosomal DEGs between 45,X vs 47,XXY in fat. **Table S27.** Autosomal DEGs between 45,X vs 46,XY in fat. **Table S28.** Autosomal DEGs between 46,XX vs 46,XY in fat. **Table S29.** Autosomal DEGs between 47,XXY vs 46,XX in fat. **Table S30.** Autosomal DEGs between 47,XXY vs 46,XY in fat. **Table S31.** Autosomal DEGs between 45,X vs 46,XX in muscle. **Table S32.** Autosomal DEGs between 45,X vs 47,XXY in muscle. **Table S33.** Autosomal DEGs between 45,X vs 46,XY in muscle. **Table S34.** Autosomal DEGs between 46,XX vs 46,XY in muscle. **Table S35.** Autosomal DEGs between 47,XXY vs 46,XX in muscle. **Table S36.** Autosomal DEGs between 47,XXY vs 46,XY in muscle.

## Data Availability

All sequence and methylation data from this study are deposited at the EGA repository (EGAS00001006996, EGAS00001007020) and will be available upon request within the General Data Protection Regulation.

## Abbreviations

Adjp: Adjusted *p*-value
SCAs: Sex chromosome aneuploidies
TS: Turner syndrome
KS: Klinefelter syndrome
DMR: Differentially methylated region
aDEG: Autosomal differentially expressed genes
xDEG: X chromosomal differentially expressed genes
aDMP: Autosomal differentially methylated positions
xDMP: X chromosomal differentially methylated positions
Fig: Figure
RNA seq: Ribonucleic acid sequencing
Xa: Active X chromosome
Xi: Inactive X chromosome
log2FC: Log2fold change

## References

[1] Gravholt CH, Andersen NH, Conway GS, Dekkers OM, Geffner ME, Klein KO (2017). Clinical practice guidelines for the care of girls and women with Turner syndrome: proceedings from the 2016 Cincinnati International Turner Syndrome Meeting. Eur J Endocrinol.

[2] Zitzmann M, Aksglaede L, Corona G, Isidori AM, Juul A, T’Sjoen G, et al. European academy of andrology guidelines on Klinefelter Syndrome Endorsing Organization: European Society of Endocrinology. Andrology. 2021;9(1):145–67. 10.1111/andr.12909 32959490

[3] Viuff MH, Berglund A, Juul S, Andersen NH, Stochholm K, Gravholt CH (2020). Sex hormone replacement therapy in Turner syndrome: impact on morbidity and mortality. J Clin Endocrinol Metab.

[4] Jørgensen KT, Rostgaard K, Bache I, Biggar RJ, Nielsen NM, Tommerup N, et al. Autoimmune diseases in women with Turner’s syndrome. Arthritis Rheum. 2010;62(3):658–66. 10.1002/art.27270 20187158

[5] Bojesen A, Gravholt CH (2011). Morbidity and mortality in Klinefelter syndrome (47, XXY). Acta Paediatr.

[6] Skakkebæk A, Moore PJ, Pedersen AD, Bojesen A, Kristensen MK, Fedder J (2017). The role of genes, intelligence, personality, and social engagement in cognitive performance in Klinefelter syndrome. Brain Behav.

[7] Schoemaker MJ, Swerdlow AJ, Higgins CD, Wright AF, Jacobs PA (2008). United Kingdom Clinical Cytogenetics G. Mortality in women with turner syndrome in Great Britain: a national cohort study. J Clin Endocrinol Metab..

[8] Zöller B, Ji J, Sundquist J, Sundquist K (2016). High Risk of Venous Thromboembolism in Klinefelter Syndrome. J Am Heart Assoc.

[9] Viuff MH, Trolle C, Wen J, Jensen JM, Norgaard BL, Gutmark EJ (2016). Coronary artery anomalies in Turner syndrome. J Cardiovasc Comput Tomogr.

[10] Skakkebæk A, Gravholt CH, Rasmussen PM, Bojesen A, Jensen JS, Fedder J (2014). Neuroanatomical correlates of Klinefelter syndrome studied in relation to the neuropsychological profile. Neuroimage Clin.

[11] Chang S, Biltoft D, Skakkebæk A, Fedder J, Bojesen A, Bor MV (2019). Testosterone treatment and association with thrombin generation and coagulation inhibition in Klinefelter syndrome: A cross-sectional study. Thromb Res.

[12] Trolle C, Mortensen KH, Pedersen LN, Berglund A, Jensen HK, Andersen NH (2013). Long QT interval in Turner syndrome–a high prevalence of LQTS gene mutations. PLoS ONE.

[13] Trolle C, Mortensen KH, Bjerre M, Hougaard DM, Cohen A, Andersen NH (2015). Osteoprotegerin in Turner syndrome - relationship to aortic diameter. Clin Endocrinol (Oxf).

[14] Trolle C, Nielsen MM, Skakkebaek A, Lamy P, Vang S, Hedegaard J (2016). Widespread DNA hypomethylation and differential gene expression in Turner syndrome. Sci Rep.

[15] Johannsen EB, Just J, Viuff MH, Okholm TLH, Pedersen SB, Meyer Lauritsen K (2022). Sex chromosome aneuploidies give rise to changes in the circular RNA profile: A circular transcriptome-wide study of Turner and Klinefelter syndrome across different tissues. Front Genet.

[16] Patro R, Duggal G, Love MI, Irizarry RA, Kingsford C (2017). Salmon provides fast and bias-aware quantification of transcript expression. Nat Methods.

[17] Love MI, Soneson C, Hickey PF, Johnson LK, Pierce NT, Shepherd L (2020). Tximeta: Reference sequence checksums for provenance identification in RNA-seq. PLoS Comput Biol.

[18] Love MI, Huber W, Anders S (2014). Moderated estimation of fold change and dispersion for RNA-seq data with DESeq2. Genome Biol.

[19] Wickham H (2009). ggplot2: Elegant Graphics for Data Analysis.

[20] Tukiainen T, Villani AC, Yen A, Rivas MA, Marshall JL, Satija R (2017). Landscape of X chromosome inactivation across human tissues. Nature.

[21] Wu T, Hu E, Xu S, Chen M, Guo P, Dai Z (2021). clusterProfiler 4.0: a universal enrichment tool for interpreting omics data. Innovation..

[22] Ashburner M, Ball CA, Blake JA, Botstein D, Butler H, Cherry JM (2000). Gene ontology: tool for the unification of biology. The Gene Ontology Consortium. Nat Genet.

[23] The Gene Ontology Consortium. The Gene Ontology resource: enriching a GOld mine. Nucleic Acids Res. 2021;49(D1):D325-34. 10.1093/nar/gkaa1113 PMC7779012 33290552

[24] Langfelder P, Horvath S (2008). WGCNA: an R package for weighted correlation network analysis. BMC Bioinformatics.

[25] Fortin J-P, Triche TJ, Hansen KD (2016). Preprocessing, normalization and integration of the Illumina HumanMethylationEPIC array with minfi. Bioinformatics.

[26] Fortin J-P, Labbe A, Lemire M, Zanke BW, Hudson TJ, Fertig EJ (2014). Functional normalization of 450k methylation array data improves replication in large cancer studies. Genome Biol.

[27] Ritchie ME, Phipson B, Wu D, Hu Y, Law CW, Shi W (2015). limma powers differential expression analyses for RNA-sequencing and microarray studies. Nucleic Acids Res..

[28] Peters TJ, Buckley MJ, Statham AL, Pidsley R, Samaras K, Lord RV (2015). De novo identification of differentially methylated regions in the human genome. Epigenetics Chromatin..

[29] Skakkebæk A, Nielsen MM, Trolle C, Vang S, Hornshøj H, Hedegaard J (2018). DNA hypermethylation and differential gene expression associated with Klinefelter syndrome. Sci Rep.

[30] Sharma A, Jamil MA, Nuesgen N, Schreiner F, Priebe L, Hoffmann P (2015). DNA methylation signature in peripheral blood reveals distinct characteristics of human X chromosome numerical aberrations. Clin Epigenetics.

[31] Balaton BP, Cotton AM, Brown CJ (2015). Derivation of consensus inactivation status for X-linked genes from genome-wide studies. Biol Sex Differ.

[32] Carrel L, Willard HF (2005). X-inactivation profile reveals extensive variability in X-linked gene expression in females. Nature.

[33] Balaton BP, Brown CJ (2021). Contribution of genetic and epigenetic changes to escape from X-chromosome inactivation. Epigenetics Chromatin.

[34] Zhang X, Hong D, Ma S, Ward T, Ho M, Pattni R (2020). Integrated functional genomic analyses of Klinefelter and Turner syndromes reveal global network effects of altered X chromosome dosage. Proc Natl Acad Sci.

[35] Raznahan A, Parikshak NN, Chandran V, Blumenthal JD, Clasen LS, Alexander-Bloch AF (2018). Sex-chromosome dosage effects on gene expression in humans. Proc Natl Acad Sci USA.

[36] Jjingo D, Conley AB, Yi SV, Lunyak VV, Jordan IK (2012). On the presence and role of human gene-body DNA methylation. Oncotarget.

[37] Hellman A, Chess A (2007). Gene body-specific methylation on the active X chromosome. Science.

[38] Cotton AM, Lam L, Affleck JG, Wilson IM, Peñaherrera MS, McFadden DE (2011). Chromosome-wide DNA methylation analysis predicts human tissue-specific X inactivation. Hum Genet.

[39] Rakyan VK, Down TA, Thorne NP, Flicek P, Kulesha E, Gräf S (2008). An integrated resource for genome-wide identification and analysis of human tissue-specific differentially methylated regions (tDMRs). Genome Res.

[40] Jaenisch R, Bird A (2003). Epigenetic regulation of gene expression: how the genome integrates intrinsic and environmental signals. Nat Genet.

[41] Rao E, Weiss B, Fukami M, Rump A, Niesler B, Mertz A (1997). Pseudoautosomal deletions encompassing a novel homeobox gene cause growth failure in idiopathic short stature and Turner syndrome. Nat Genet.

[42] Ottesen AM, Aksglaede L, Garn I, Tartaglia N, Tassone F, Gravholt CH (2010). Increased number of sex chromosomes affects height in a nonlinear fashion: a study of 305 patients with sex chromosome aneuploidy. Am J Med Genet A.

[43] Marchini A, Rappold G, Schneider KU (2007). SHOX at a glance: from gene to protein. Arch Physiol Biochem.

[44] Clement-Jones M, Schiller S, Rao E, Blaschke RJ, Zuniga A, Zeller R (2000). The short stature homeobox gene SHOX is involved in skeletal abnormalities in Turner syndrome. Hum Mol Genet.

[45] Landen S, Jacques M, Hiam D, Alvarez-Romero J, Harvey NR, Haupt LM (2021). Skeletal muscle methylome and transcriptome integration reveals profound sex differences related to muscle function and substrate metabolism. Clin Epigenetics.

[46] Bojesen A, Høst C, Gravholt CH. Klinefelter’s syndrome, type 2 diabetes and the metabolic syndrome: the impact of body composition. Mol Hum Reprod. 2010;16(6):396–401. 10.1093/molehr/gaq016 20231162

[47] Gravholt CH, Hjerrild BE, Mosekilde L, Hansen TK, Rasmussen LM, Frystyk J (2006). Body composition is distinctly altered in Turner syndrome: relations to glucose metabolism, circulating adipokines, and endothelial adhesion molecules. Eur J Endocrinol.

[48] De Bonis ML, Cerase A, Matarazzo MR, Ferraro M, Strazzullo M, Hansen RS (2006). Maintenance of X- and Y-inactivation of the pseudoautosomal (PAR2) gene SPRY3 is independent from DNA methylation and associated to multiple layers of epigenetic modifications. Hum Mol Genet.

[49] Vergult S, Dheedene A, Meurs A, Faes F, Isidor B, Janssens S (2015). Genomic aberrations of the CACNA2D1 gene in three patients with epilepsy and intellectual disability. Eur J Human Genet EJHG.

[50] Kessi M, Chen B, Peng J, Yan F, Yang L, Yin F (2021). Calcium channelopathies and intellectual disability: a systematic review. Orphanet J Rare Dis.

[51] Burashnikov E, Pfeiffer R, Barajas-Martinez H, Delpón E, Hu D, Desai M (2010). Mutations in the cardiac L-type calcium channel associated with inherited J-wave syndromes and sudden cardiac death. Heart Rhythm.

[52] Viuff MH, Stochholm K, Juul S, Gravholt CH. Disorders of the eye, ear, skin, and nervous system in women with Turner syndrome -a nationwide cohort study. Eur J Hum Genet. 2022;30:228-36. 10.1038/s41431-021-00989-5 PMC8821537 34707298

[53] Hobkirk R (1985). Steroid sulfotransferases and steroid sulfate sulfatases: characteristics and biological roles. Can J Biochem Cell Biol.

[54] Valigora SD, Lib PK, Dunphy G, Turner M, Ely DL (2000). Steroid sulfatase inhibitor alters blood pressure and steroid profiles in hypertensive rats. J Steroid Biochem Mol Biol.

[55] Wen J, Trolle C, Viuff MH, Ringgaard S, Laugesen E, Gutmark EJ (2018). Impaired aortic distensibility and elevated central blood pressure in Turner syndrome: a cardiovascular magnetic resonance study. J Cardiovasc Magn Reson.

[56] Boucher J, Masri B, Daviaud D, Gesta S, Guigné C, Mazzucotelli A (2005). Apelin, a newly identified adipokine up-regulated by insulin and obesity. Endocrinology.

[57] Sinitsky MY, Dyleva YA, Uchasova EG, Belik EV, Yuzhalin AE, Gruzdeva OV (2022). Adipokine gene expression in adipocytes isolated from different fat depots of coronary artery disease patients. Arch Physiol Biochem.

[58] Yan L, Jin W, Zhao Q, Cui X, Shi T, Xu Y (2021). PWWP2B fine-tunes adipose thermogenesis by stabilizing HDACs in a NuRD subcomplex. Adv Sci (Weinh)..

[59] Kaushansky K, Broudy VC, Lin N, Jorgensen MJ, McCarty J, Fox N (1995). Thrombopoietin, the Mp1 ligand, is essential for full megakaryocyte development. Proc Natl Acad Sci USA.

[60] Viuff MH, Stochholm K, Grønbaek H, Berglund A, Juul S, Gravholt CH (2021). Increased occurrence of liver and gastrointestinal diseases and anaemia in women with Turner syndrome - a nationwide cohort study. Aliment Pharmacol Ther.

[61] Schweighofer N, Haudum CW, Schmidt A, Kolesnik E, Colantonio CR, Pieske B, Pieber TR, Obermayer-Pietsch B (2019). Diabetes-, sex-, and BMI specific associations of genetic variants in PRRG1 with cardiovascular surrogates in a large cohort at CV risk. 21st European Congress of Endocrinology.

[62] SLC16A2 solute carrier family 16 member 2 [Homo sapiens (human)]. 2022. Available from: https://www.ncbi.nlm.nih.gov/gene/6567.

[63] Sarret C, Oliver Petit I, Tonduti D, et al. Allan-Herndon-Dudley Syndrome. In: Adam MP, Ardinger HH, Pagon RA, Wallace SE, Bean LJH, Gripp KW, et al., editors. GeneReviews(®). Seattle: University of Washington, Seattle; 1993. (Copyright © 1993-2022). 20301789

[64] Cox TC, Allen LR, Cox LL, Hopwood B, Goodwin B, Haan E (2000). New mutations in MID1 provide support for loss of function as the cause of X-linked Opitz syndrome. Hum Mol Genet.

[65] Robin NH, Opitz JM, Muenke M (1996). Opitz G/BBB syndrome: clinical comparisons of families linked to Xp22 and 22q, and a review of the literature. Am J Med Genet.

[66] Mortensen KH, Andersen NH, Gravholt CH (2012). Cardiovascular phenotype in Turner syndrome–integrating cardiology, genetics, and endocrinology. Endocr Rev.

[67] Lan F, Bayliss PE, Rinn JL, Whetstine JR, Wang JK, Chen S (2007). A histone H3 lysine 27 demethylase regulates animal posterior development. Nature.

[68] Mansour AA, Gafni O, Weinberger L, Zviran A, Ayyash M, Rais Y (2012). The H3K27 demethylase Utx regulates somatic and germ cell epigenetic reprogramming. Nature.

[69] Lindgren AM, Hoyos T, Talkowski ME, Hanscom C, Blumenthal I, Chiang C (2013). Haploinsufficiency of KDM6A is associated with severe psychomotor retardation, global growth restriction, seizures and cleft palate. Hum Genet.

[70] Piton A, Gauthier J, Hamdan FF, Lafrenière RG, Yang Y, Henrion E (2011). Systematic resequencing of X-chromosome synaptic genes in autism spectrum disorder and schizophrenia. Mol Psychiatry.

[71] Pena JLB, Santos WC, Siqueira MHA, Sampaio IH, Moura ICG, Sternick EB (2021). Glycogen storage cardiomyopathy (PRKAG2): diagnostic findings of standard and advanced echocardiography techniques. Eur Heart J Cardiovasc Imaging.

[72] Winder WW (2001). Energy-sensing and signaling by AMP-activated protein kinase in skeletal muscle. J Appl Physiol (1985)..

[73] Hu J, Tang B, Wang J, Huang K, Wang Y, Lu S (2020). Familial atrial enlargement, conduction disorder and symmetric cardiac hypertrophy are early signs of PRKAG2 R302Q. Curr Med Sci.

[74] Beyzaei Z, Ezgu F, Geramizadeh B, Alborzi A, Shojazadeh A (2021). Novel PRKAG2 variant presenting as liver cirrhosis: report of a family with 2 cases and review of literature. BMC Med Genomics.

