# Supplementary material for: X chromosome dosage and the genetic impact across human tissues
Source: Genome Med. 2023 Mar 28;15:21. doi: 10.1186/s13073-023-01169-4 (PMC10053618; doi:10.1186/s13073-023-01169-4)
Supplement: Supplementary file 1 — Additional file 1: Figure S1. Manhattan plots of X chromosomal differentially expressed genes. Figure S2. Venn diagrams of X chromosomal differentially expressed genes. Figure S3. Circular plots of PAR genes. Figure S4. Manhattan plots of autosomal differentially expressed genes. Figure S5. Densityplot of autosomal differentially expressed genes (aDEGs). Figure S6. Venn diagrams of autosomal differentially expressed genes. Figure S7. WGCNA analysis module 1–3. Figure S8. WGCNA analysis trait 1–3. Figure S9. WGCNA analysis module 4–6. Figure S10. WGCNA analysis trait 4–5. Figure S11. Venn diagrams of X chromosomal and autosomal differentially methylated sites. Figure S12. Venn diagram of X chromosomal and autosomal differentially methylated sites. Figure S13. Distribution of X chromosomal differentially methylated sites (xDMPs) and autosomal differentially methylated sites (aDMPs) between different contrasts in relation to genic region. Figure S14. Correlation plots of significant differentially methylated positions (DMPs) in correlation with gene expression. Figure S15. Correlation plots of significantly differentially methylated regions (DMRs) in correlation with gene expression. [file 13073_2023_1169_MOESM1_ESM.pdf]

Additional file 1

X chromosome dosage and the genetic impact across human tissues

Mette Viuff[a,b,c,1,*] Anne Skakkebæk[a,c,d,1,*], Emma B. Johannsen[a,c], Simon Chang[b], Steen Bønlykke Pedersen[b], Katrine Meyer Lauritsen[b], Mette Glavind Bülow Pedersen[b], Christian Trolle[b], Jesper Just[a,c], Claus H. Gravholt[a,b,c,2]

[1]These authors contributed equally to this work

[*]MD Mette H. Viuff, Department of Molecular Medicine and Department of Internal Medicine, Endocrinology and Department of Clinical Medicine, Aarhus University Hospital, Palle-Juul Jensens Boulevard 99, 8200 Aarhus N, Denmark. Phone: 004523705085.

*MD PhD Anne Skakkebæk. Department of Clinical Genetics, Department of Molecular Medicine and Department of Clinical Medicine, Aarhus University Hospital, Palle-Juul Jensens Boulevard 99, 8200 Aarhus N, Denmark. Phone: 004527212998.

Email: metteviuff@clin.au.dk: asj@clin.au.dk

This PDF file includes:

Supplementary figures S1 to S15

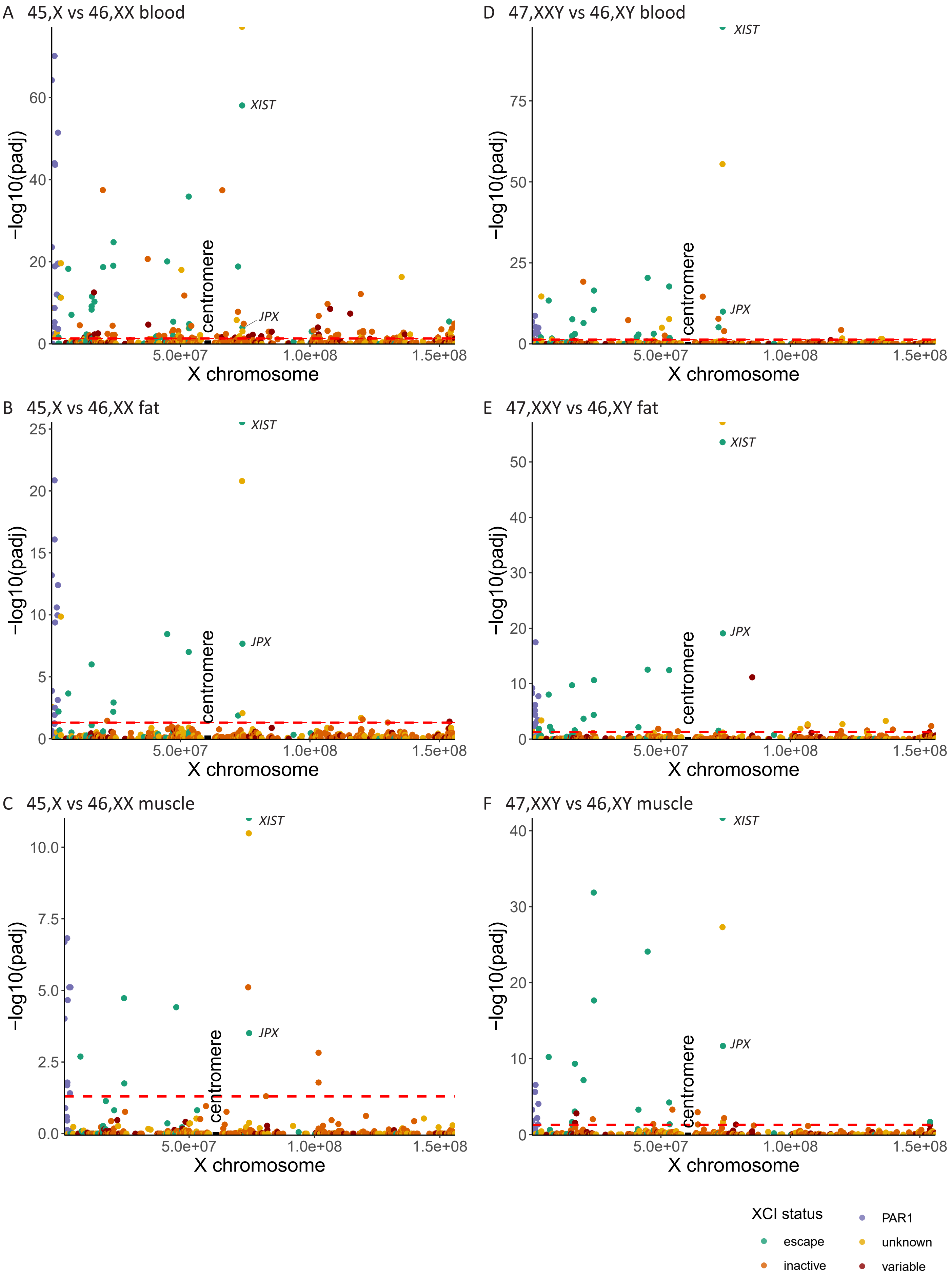

Figure S1. Manhattan plots of X chromosomal differentially expressed genes.
Manhattanplot of X chromosomal differentially expressed genes (xDEGs) between 45,X and 46,XX (A, B, C) and between 47,XXY and 46,XY (D, E, F) in blood, fat and muscle. Red line represents padj = 0.05.

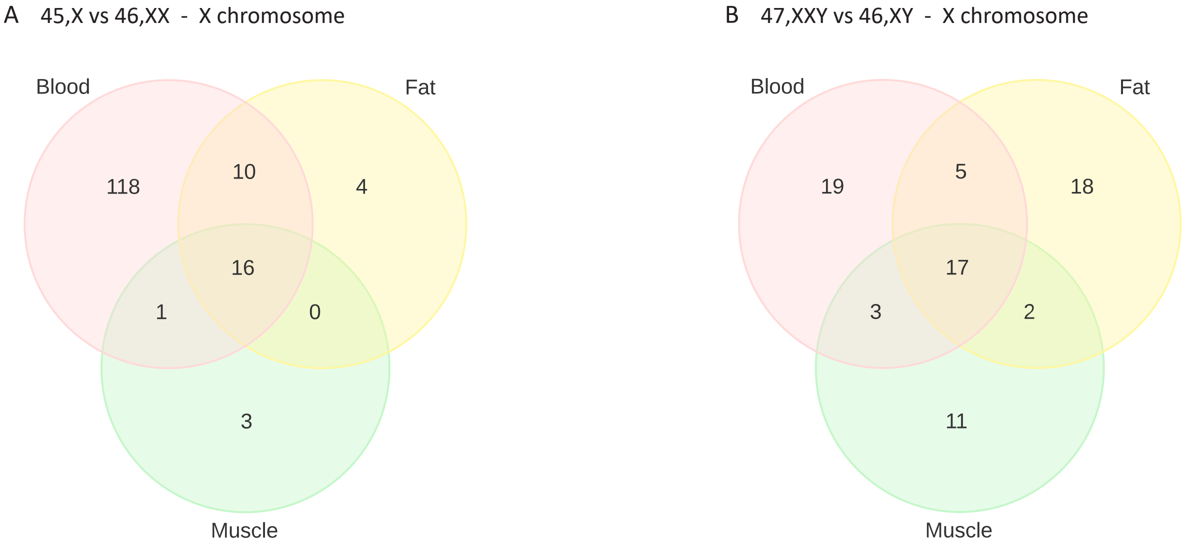

Figure S2. Venn diagrams of X chromosomal differentially expressed genes.
Venn diagrams illustrate overlapping X chromosomal differentially expressed genes in blood, fat and muscle comparing A) 45,X vs 46,XX and B) 47,XXY vs 46,XY.

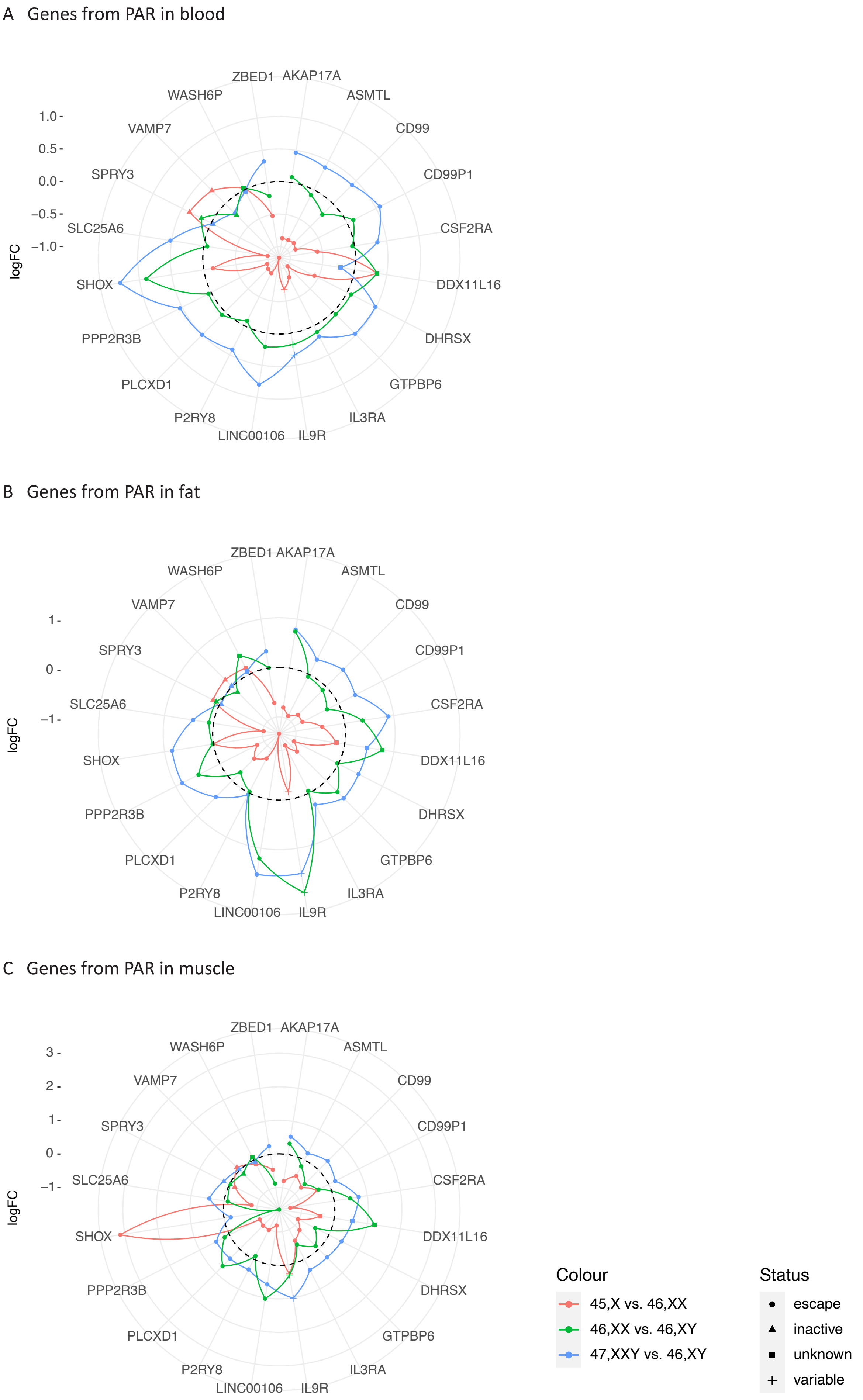

Figure S3. Circular plots of PAR genes.
Circular plots of PAR genes in blood(A), fat(B) and muscle(C).

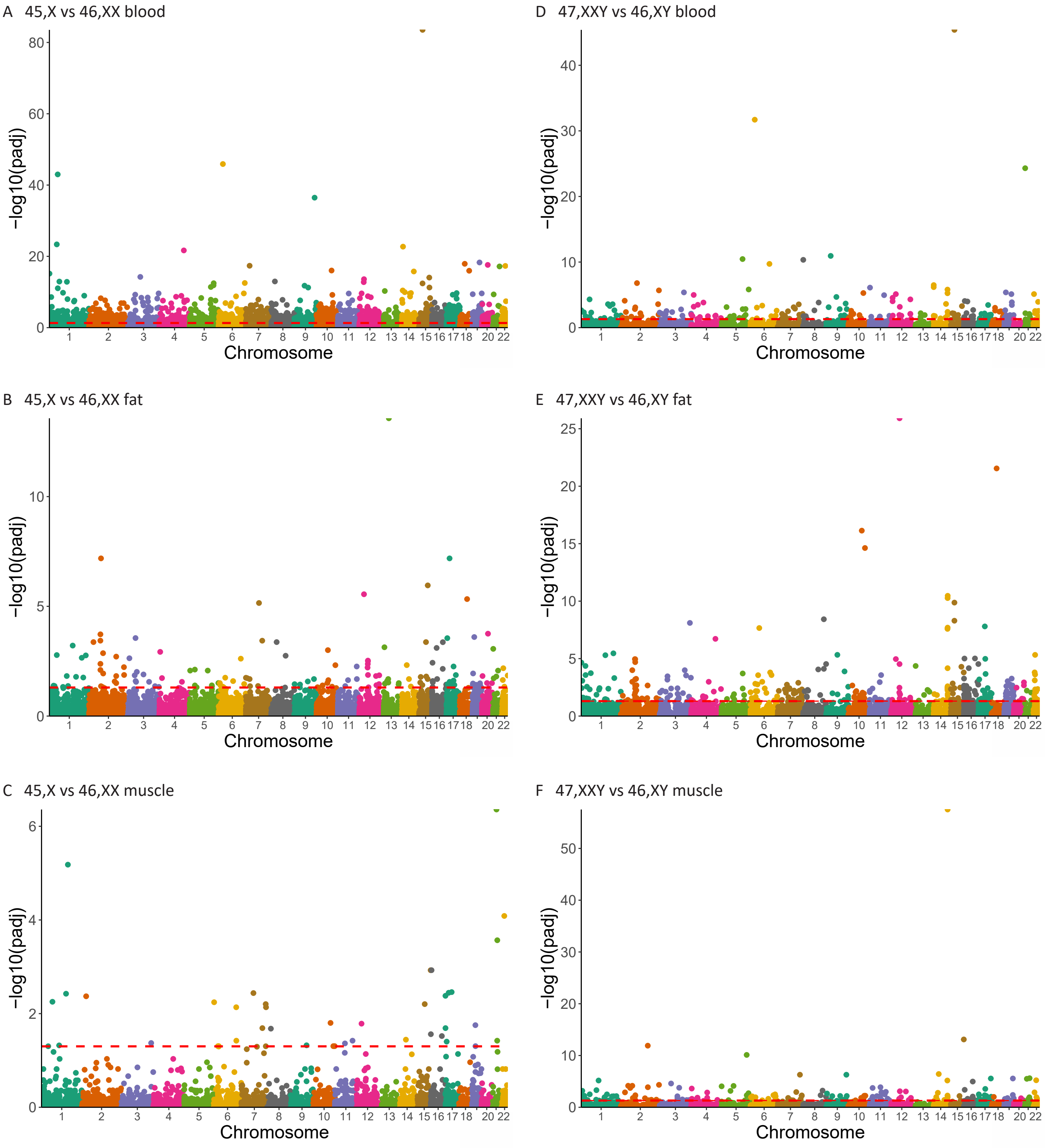

Figure S4. Manhattan plots of autosomal differentially expressed genes.
Manhattanplot of autosomal differentially expressed genes (aDEGs) between 45,X and 46,XX (A, B,C) and between 47,XXY and 46,XY (D, E, F) in blood, fat and muscle. Red line represents padj = 0.05.

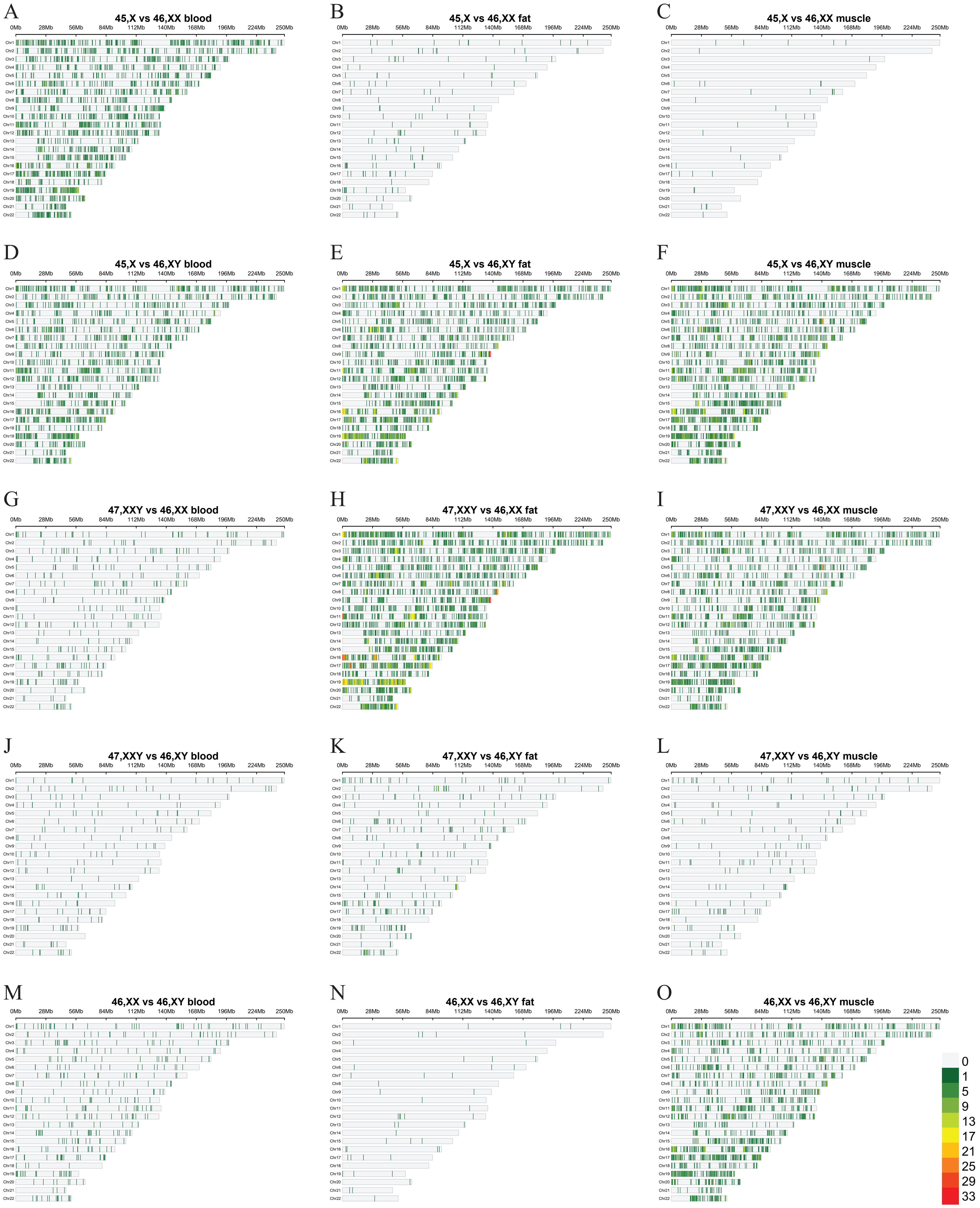

Figure S5. Densityplot of autosomal differentially expressed gene (aDEG).

Densityplot of autosomal differentially expressed gene (aDEG) illustrating chromosome wise the number of aDEGs within 1 Mb window size (padj<0.05, abs. log-2FC≥0) between karyotypes in blood, fat and muscle. (A) aDEGs in blood between 45,X and 46,XX. (B) aDEGs in fat tissue between 45,X and 46,XX. (C) aDEGs in muscle tissue between 45,X and 46,XX. (D) aDEGs in blood between 45,X and 46,XY. (E) aDEGs in fat tissue between 45,X and 46,XY. (F) aDEGs in muscle tissue between 45,X and 46,XY. (G) aDEGs in blood between 47,XXY and 46,XX. (H) aDEGs in fat tissue between 47,XXY and 46,XX. (I) aDEGs in muscle tissue between 47,XXY and 46,XX. (J) aDEGs in blood between 47,X XY and 46,XY. (K) aDEGs in fat tissue between 47,XXY and 46,XY. (L) aDEGs in muscle tissue between 47,XXY and 46,XY. (M) aDEGs in blood between 46,XX and 46,XY. (N) aDEGs in fat tissue between 46,XX and 46,XY. (O) aDEGs in muscle tissue between 46,XX and 46,XY.

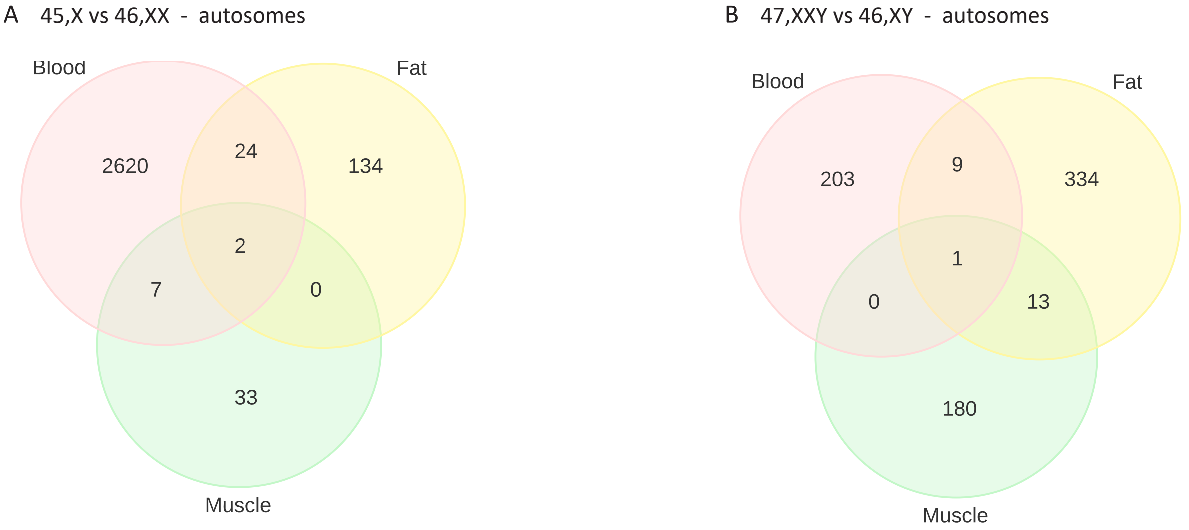

Figure S6. Venn diagrams of autosomal differentially expressed genes.
Venn diagrams illustrate overlapping autosomal differentially expressed genes in blood, fat and muscle comparing A) 45,X vs 46,XX and B) 47,XXY vs 46,XY.

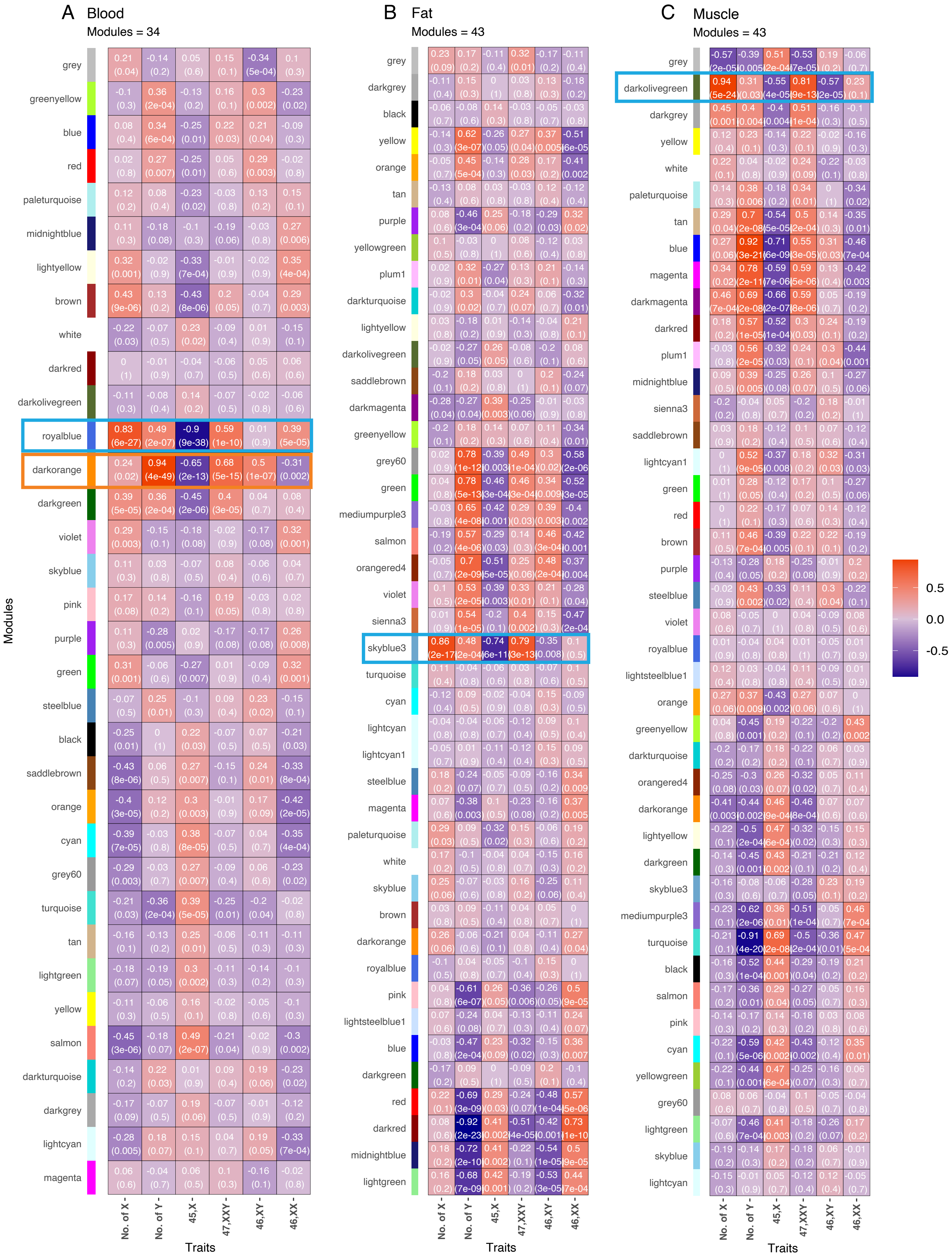

Figure S7. WGCNA analysis module 1-3.
Weighted gene co-expression network analysis of all genes, correlating modules of co-expressed genes in blood (A), fat (B) and muscle (C) to the number of X chromosomes, number of Y chromosomes, TS, KS, males and females of normal karyotype. Colors correspond to module-trait correlations, positive marked in red and negative in blue. Colored frames indicate modules with strong correlations to the number of X chromosomes (blue) and Y chromosomes (orange).

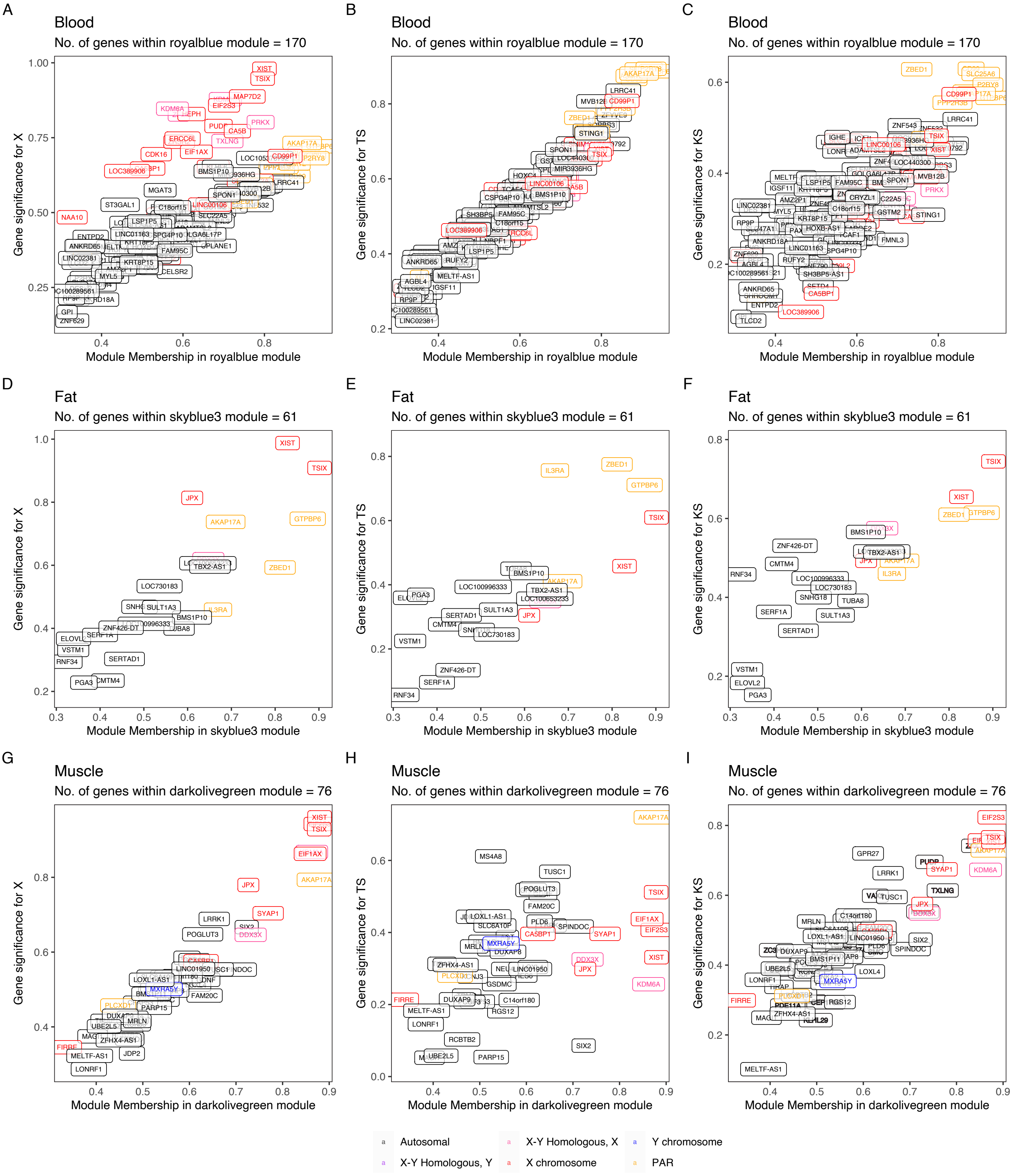

Figure S8. WGCNA analysis trait 1-3.
The module membership for genes within modules highly correlated to the number of X chromosomes in blood (A-C), fat (D-F) and muscle (G-I) based on all genes, vs. trait significance for the number of X chromosomes (A, D, G), 47,XXY (B, E, H) and 45,X (C, F, I). Genes are grouped by chromosomal origin and location (autosomal, X-Y homologous X, X-Y homologous Y, Y chromosome, X chromosome, PAR) as presented in the legend.

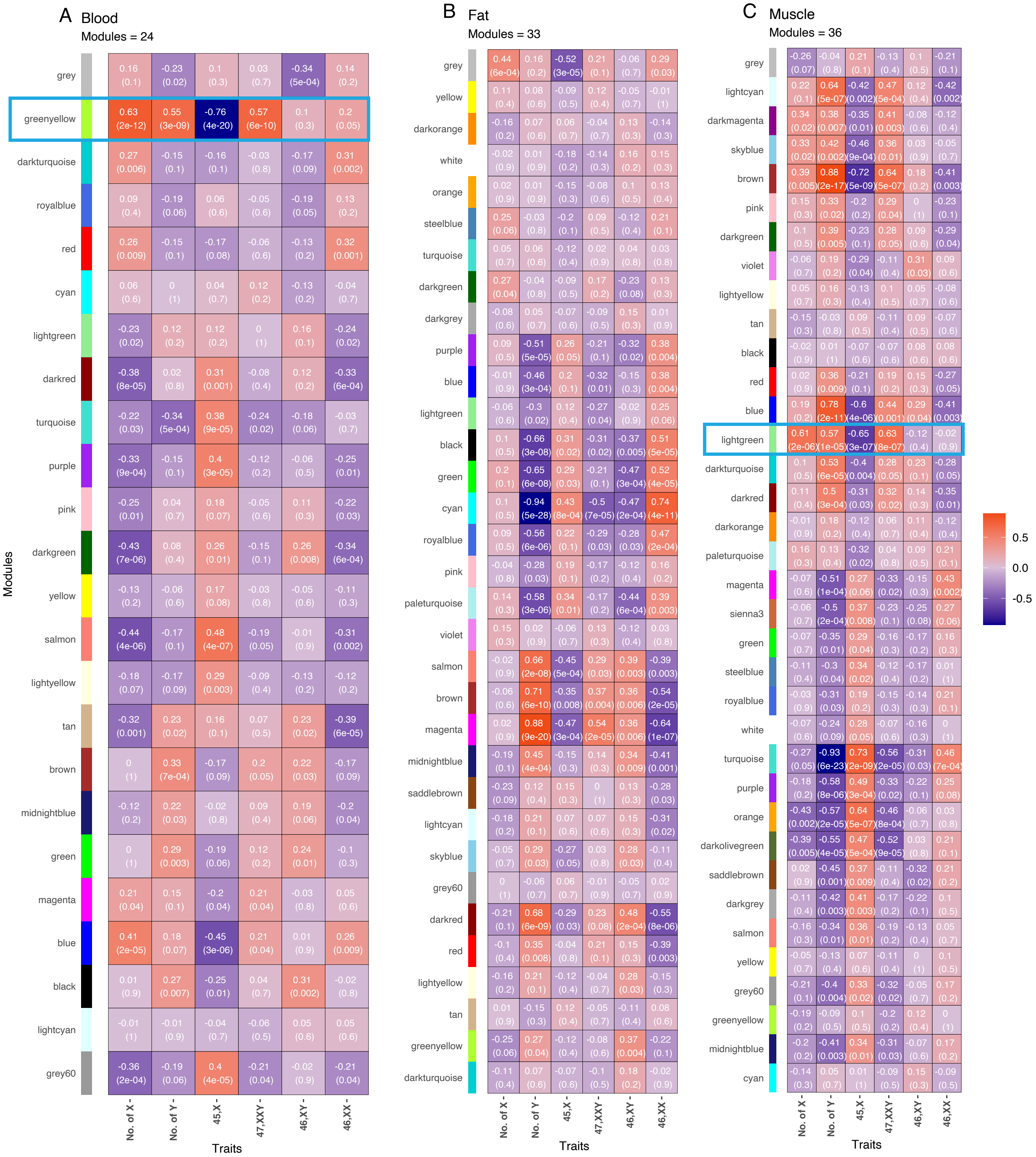

Figure S9. WGCNA analysis module 4-6.
Weighted gene co-expression network analysis of autosomal genes, correlating modules of co-expressed genes in blood (1), fat (2) and muscle (3) to the number of X chromosomes, number of Y chromosomes, 45,X, 47,XXY, males and females of normal karyotype. Colors correspond to module-trait correlations, positive marked in red and negative in blue. Blue frames indicate modules with strong correlations to the number of X chromosomes.

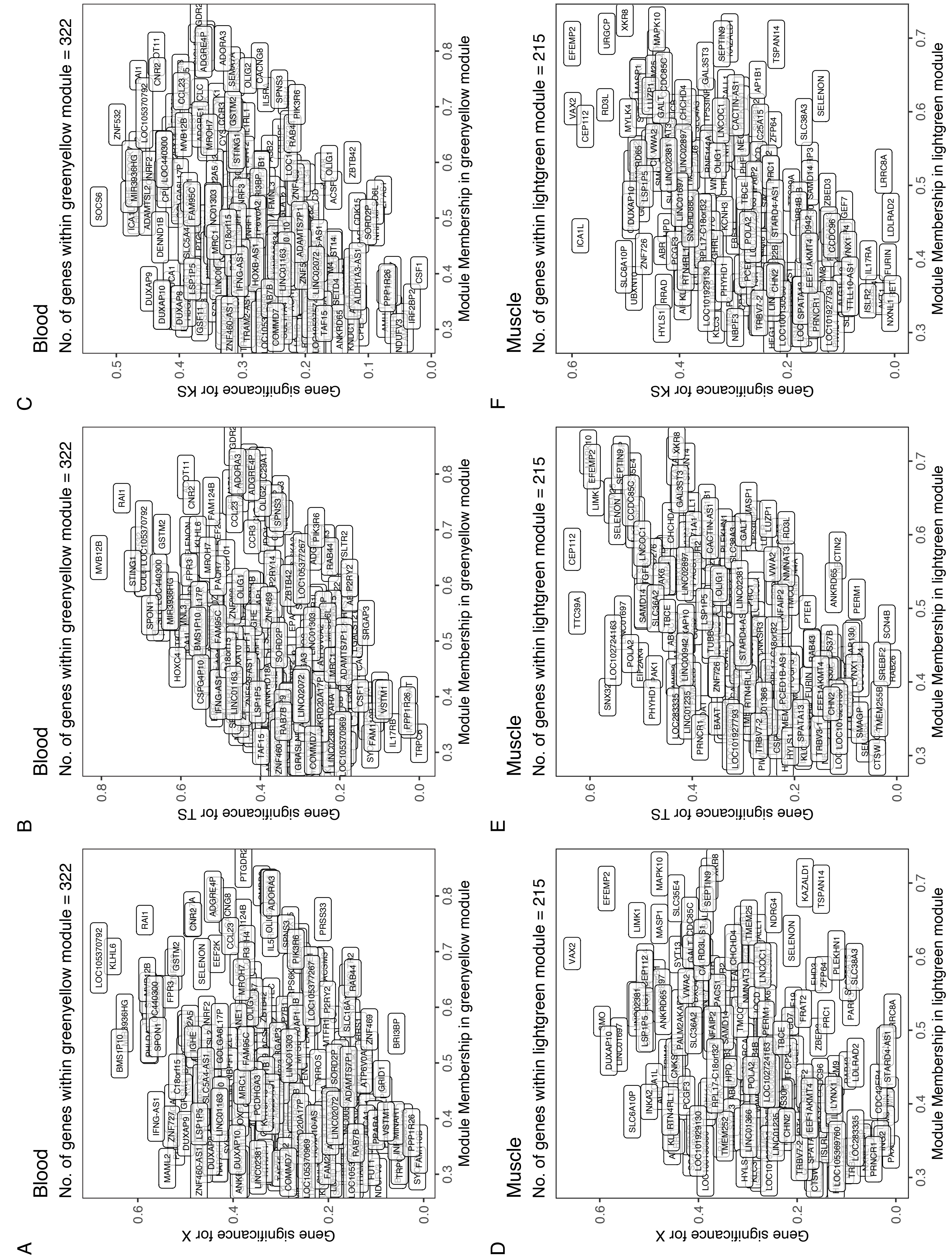

Figure S10. WGCNA analysis trait 4-5.
The module membership for genes within modules highly correlated to the number of X chromosomes in blood (A-C) and muscle (D-F) based on autosomes, vs. trait significance for the number of X chromosomes (A, D), 47,XXY (B, E) and 45,X (C, F).

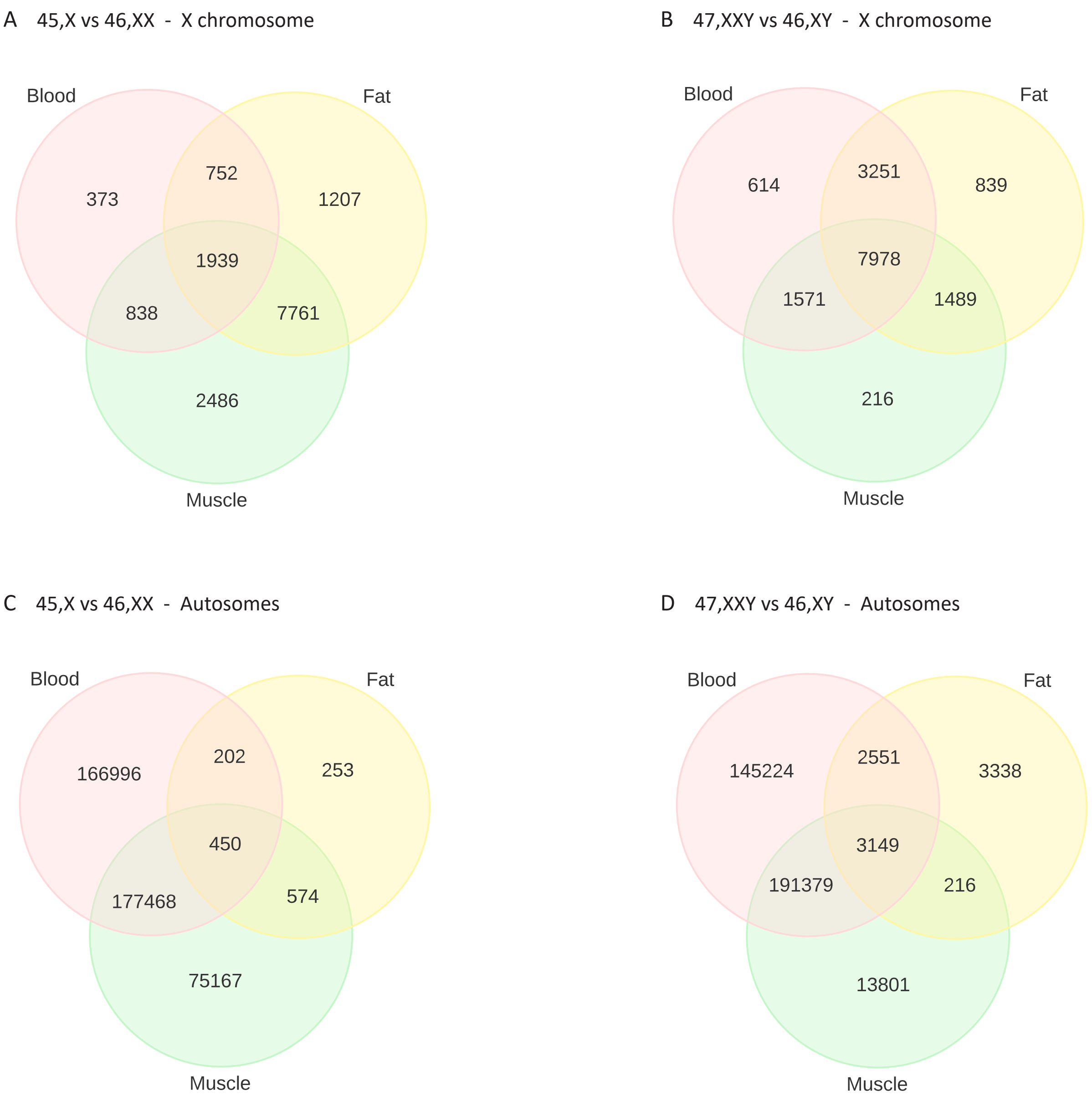

Figure S11. Venn diagrams of X chromosomal and autosomal differentially methylated sites. Venn diagrams illustrate overlapping X chromosomal (A, B) and autosomal (C, D) differentially methylated sites in blood, fat and muscle comparing 45,X vs 46,XX (A, C) and 47,XXY vs 46,XY (B, D).

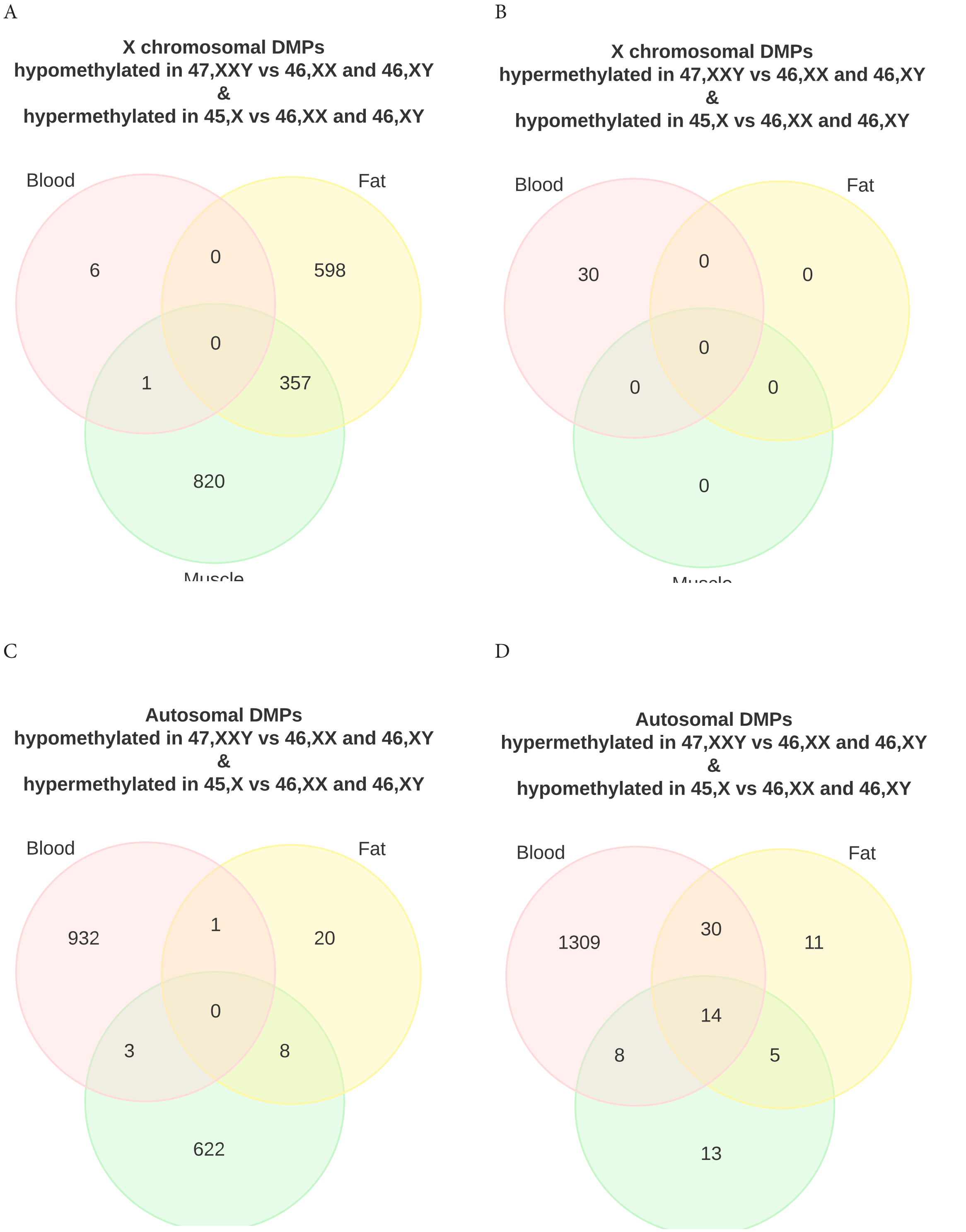

Figure S12. Venn diagram of X chromosomal and autosomal differentially methylated sites
Venn diagram of the number of (A) X chromosomal differentially methylated sites (xDMPs) in blood, fat and muscle being hypomethylated in 47,XXY and hypermethylated in 45,X compared to both 46,XX and 46,XY, (B) X chromosomal differentially methylated sites (xDMPs) in blood, fat and muscle being hypermetylated in 47,XXY and hypomethylated in 45,X compared to both 46,XX and 46,XY, (C) autosomal differentially methylated sites (aDMPs) in blood, fat and muscle being hypomethylated in 47,XXY and hypermethylated in 45,X compared to both 46,XX and 46,XY and46,XY, (D) autosomal differentially methylated sites (aDMPs) in blood, fat and muscle be ing hypermethylated in 47,XXY and hypomethylated in 45,X compared to both 46,XX and 46,XY.

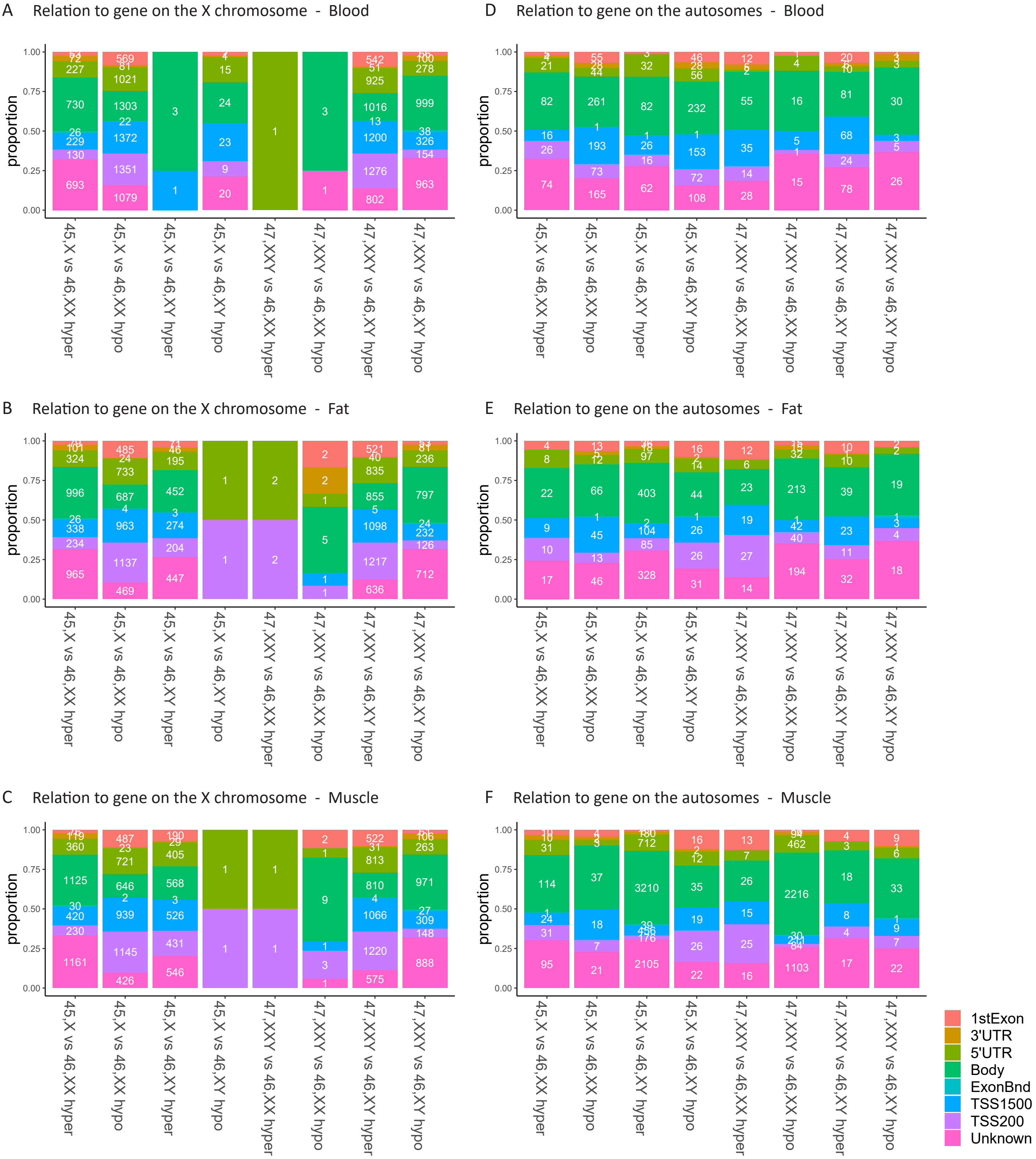

Figure S13. Distribution of X chromosomal differentially methylated sites (xDMPs) and autosomal differentially methylated sites (aDMPs) between different contrasts in relation to genic region. The white numbers in the barplot refer to the number of DMPs in the specific genic region. "Hyper" refers to the number of hypermethylated DMPs in the given comparison, while "hypo" refers to the number of hypomethylated DMPs. (A) xDMPs in blood, (B) xDMPs in fat, (C) xDMPs in muscle (D) aDMPs in blood, (E) aDMPs in fat, and (F) aDMPs in muscle.

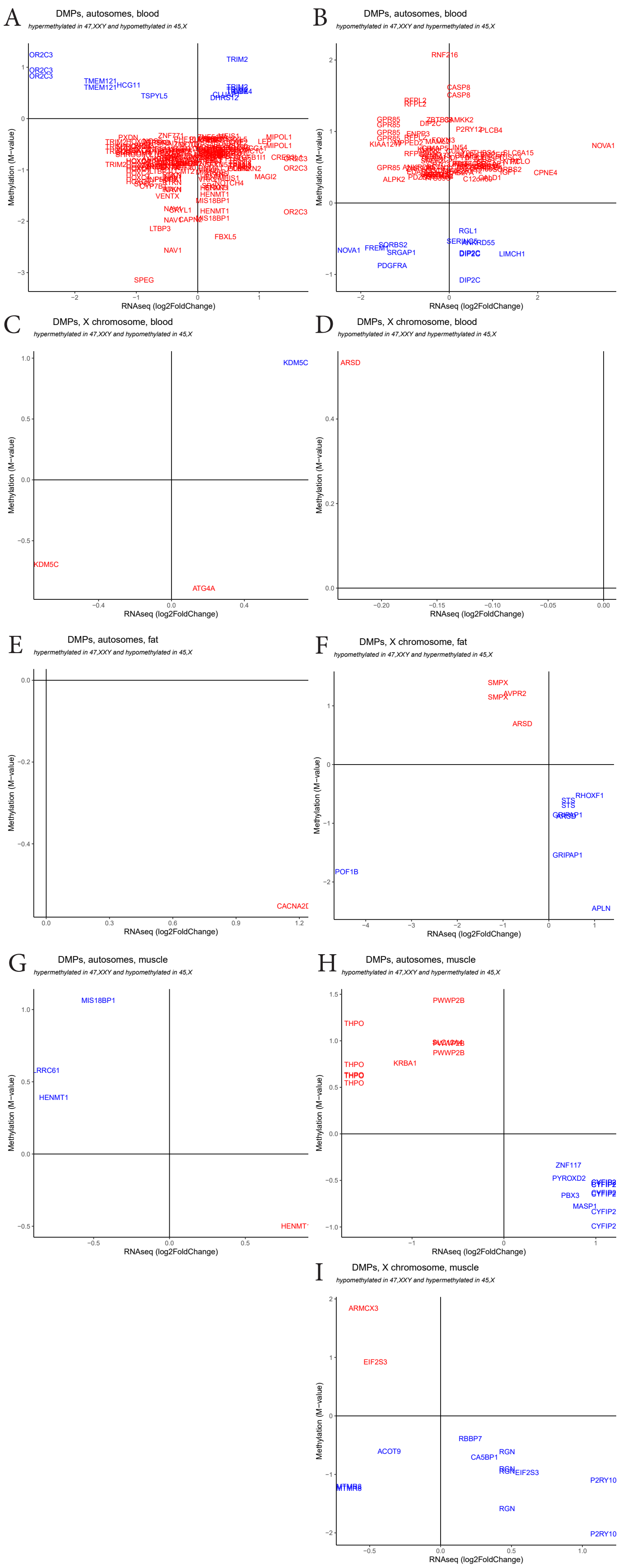

Figure S14. Correlation plots of significant differentially methylated positions (DMPs) in correlation with gene expression.

Correlation plots demonstrating all significant differentially methylated positions (DMPs) both hypermethylated in 47,XXY and hypomethylated in 45,X or vice versa correlated with significant differential gene expression analysis of autosomal DMPs in blood (A, B), X chromosomal DMPs in blood (C, D) autosomal DMPs in fat (E), X chromosomal DMPs in fat (F), autosomal DMPs in muscle (G, H), X chromosomal DMPs in muscle (I). Genes names written in blue indicate genes which are differentially expressed in 47,XXY vs. 46,XY, while gene names written in red indicate genes differentially expressed in 45,X vs. 46,XX.

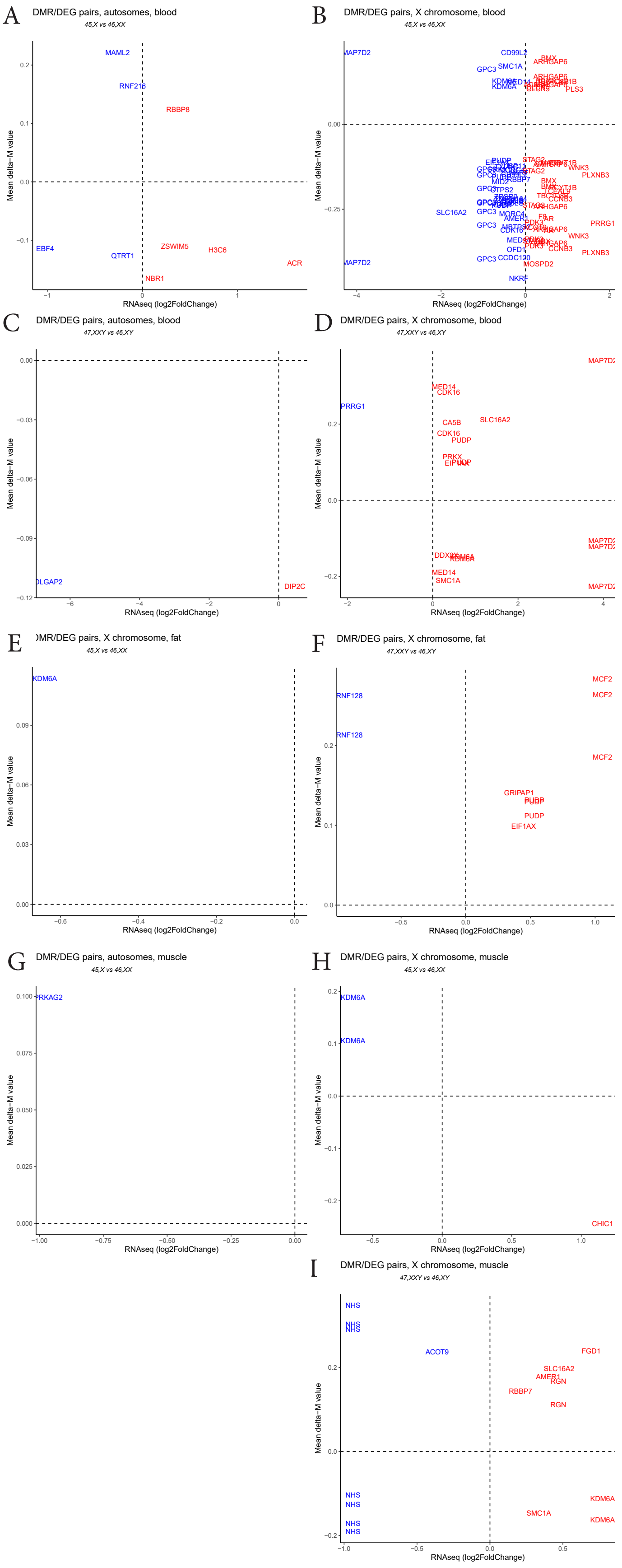

Figure S15. Correlation plots of significantly differentially methylated regions (DMRs) in correlation with gene expression.

Correlation plots of significantly autosomal differentially methylated regions (aDMRs) in correlation with significant differential gene expression in 45,X vs. 46,XX in blood (A) and muscle (G) and in 47,XXY vs. 46,XY in blood (C). Correlation plots of significantly X chromosomal DMRs (xDMRs) in correlation with significant differential gene expression in 45,X vs. 46,XX in blood (B), fat (E) and muscle (H) and in 47,XXY vs. 46,XY in blood(D), fat (F) and muscle (I). The color blue represents downregulated genes, while red represents upregulated genes. The Y axis represents the M-value and the X axis represents the log2FoldChange. XIST is not plotted due to scaling.
