# Supplementary material for: X chromosome dosage and the genetic impact across human tissues
Source: Genome Med. 2023 Mar 28;15:21. doi: 10.1186/s13073-023-01169-4 (PMC10053618; doi:10.1186/s13073-023-01169-4)
Supplement: Supplementary file 2 — Additional file 2: Text S1. Expression profile of escape genes in fat and muscle. Text S2. Enrichment analysis of DEGs. Text S3. Correlation between DMPs and gene expression. Text S4. Correlation between DMRs and DEGs across tissues. [file 13073_2023_1169_MOESM2_ESM.pdf]

Additional file 2

X chromosome dosage and the genetic impact across human tissues

Mette Viuff[a,b,c,1,*]Anne Skakkebæk[a,c,d,1,*], Emma B. Johannsen[a,c], Simon Chang[b], Steen Bønlykke Pedersen[b], Katrine Meyer Lauritsen[b], Mette Glavind Bülow Pedersen[b], Christian Trolle[b], Jesper Just[a,c], Claus H. Gravholt[a,b,c,2]

[1]These authors contributed equally to this work

[*]MD Mette H. Viuff, Department of Molecular Medicine and Department of Internal Medicine, Endocrinology and Department of Clinical Medicine, Aarhus University Hospital, Palle-Juul Jensens Boulevard 99, 8200 Aarhus N, Denmark. Phone: 004523705085.

*MD PhD Anne Skakkebæk. Department of Clinical Genetics, Department of Molecular Medicine and Department of Clinical Medicine, Aarhus University Hospital, Palle-Juul Jensens Boulevard 99, 8200 Aarhus N, Denmark. Phone: 004527212998.

Email: metteviuff@clin.au.dk; asj@clin.au.dk

This PDF file includes: Supplementary results

Supplementary text S1 to S4

Text S1.

Expression profile of escape genes in fat and muscle. In fat and muscle, less than half of the escape genes were differentially expressed in 45,X vs. 46,XX (Fat: 19 out of 58; Muscle: 14 out of 53) and 47,XXY vs. 46,XY (Fat: 23 out of 58; Muscle: 19 out of 53). We observed a similar expression pattern as in blood with genes being downregulated in 45,X vs. 46,XX and upregulated in 47,XXY vs. 46,XY.

Text S2

Enrichment analysis of DEGs.

Analysis of DEGs found in blood between 45,X and 46,XX (n=2554) revealed enrichment primary for biological processes (GOBP) related to the immune system but also to coagulation. Enrichment for disease association (DisGENet) revealed enrichment for terms related to inflammatory diseases, congenital malformation and dysmorphism and neurodegenerative disorders (Fig. 4). Based on the 174 DEGs in fat tissue, enrichments were seen for GOBP terms related to demethylation, whereas enrichment for disease association (DisGENet) revealed enrichment for terms related to sex chromosome aneuploidies. No enrichments were significant based on the 62 DEGs found in muscle tissue. Analysis of DEGs in blood between 47,XXY and 46,XY (n=216) revealed enrichment for GOBP terms related to mRNA splicing, actomysin structure organization, development of metanephric glomerulus morphogenesis and platelet-derived growth factor receptor signaling pathways, whereas enrichment analysis of DEGs in fat between 47,XXY and 46,XY (n=353) revealed enrichment for GO term related to the immune system (Fig. 4). Enrichment for disease associations (DisGENet) based on DEGs in fat between 47,XXY and 46,XY revealed enrichment for terms related to severe intellectual disability, language disorders, abnormality of the dentition as well as different skin diseases, whereas in muscle based on 205 DEGs enrichment for terms related to hearing loss and skeletal abnormalities was seen (Fig. 4). No enrichments for disease association (DisGENet) terms were seen in blood and no significant enrichments were seen in muscle in relation to GOBP terms.

Text S3

Correlation between DMPs and gene expression

In blood, three autosomal genes were both hypermethylated in 47,XXY, hypomethylated in 45,X and at the same time DEGs (*OR2C3, TRIM2 and HCG11*) (Additional file1: Fig. S14). *OR2C3* was significantly downregulated and hypermethylated in 47,XXY and likewise upregulated and hypomethylated in 45,X. Differential methylation of *OR2C3* has been proposed implicated in mild cognitive impairment (1). *TRIM2* was hypermethylated and upregulated in 47,XXY, whilst hypomethylated and downregulated in 45,X. *TRIM2* drives neurite outgrowth and polarization and plays a neuroprotective role (2).

Six autosomal genes were both hypomethylated in 47,XXY and hypermethylated in 45,X and DEGs *(NOVA1, ANKRD55, DIP2C, SORBS2, SERINC5, SRGAP1*) (Additional file 1: Fig. S14). *DIP2C* was downregulated in 45,X and upregulated in 47,XXY, and variants have been reported in neuronal diseases (3), autism spectrum disorder (4), and epilepsy (5).

Two X chromosomal genes were both DEG and annotated to DMPs (Additional file 1: Fig. S14). *ARSD* was hypomethylated and upregulated in 47,XXY, while hypermethylated and downregulated in 45,X. *KDM5C* were hypermethylated and upregulated in 47,XXY, while hypomethylated and downregulated in 45,X.

In fat, *SMPX, AVPR2, POF1B, STS,* and *APLN* were hypermethylated in 45,X and hypomethylated in 47,XXY. *SMPX* and *AVPR2* were both hypomethylated in 47,XXY while hypermethylated and downregulated in 45,X (Additional file 1: Fig. S14). *SMPX* deficiency has been implicated in progressive hearing loss in mice (6). Similarly sensorineural hearing loss is

frequent in 45,X (7). The *AVPR2* gene is involved in production of the vasopressin V2 receptor (8). *POF1B* was hypomethylated in 47,XXY and considerably downregulated in 47,XXY (log2FC = -4.4), while *STS* and *APLN* were hypomethylated and upregulated in 47,XXY.
In muscle, *THPO* was hypermethylated and downregulated in 45,X vs. 46,XX, while hypomethylated in 47,XXY vs. 46,XY (Additional file 1: Fig. S14). *PWWP2B* was hypomethylated in 47,XXY while hypermethylated and downregulated in 45,X vs. 46,XX. *CYFIP2* was hypomethylated and upregulated in 47,XXY vs. 46,XY. *MASP1* was hypomethylated and upregulated in 47,XXY vs. 46,XY. Only *EIF2S3* was significantly hypomethylated and upregulated in 47,XXY and at the same time hypermethylated and downregulated in 45,X. *EIF2S3* has previously been demonstrated upregulated in blood (9,10), and has been associated with cognition and intelligence (11).

Text S4

Correlation between DMRs and DEGs across tissues

Our analysis revealed one xDMR/xDEG´s of particular interest, KDM6A, identified as a xDMR/DEG in both 45,X vs. 46,XX and 47,XXY vs. 46,XY in both blood, fat and muscle, hypomethylated and upregulated in 47,XXY, while hypermethylated and downregulated in 45,X vs. 46,XX. In blood, 20 xDMR/DEGs (13 genes) were identified comparing 47,XXY vs. 46,XY. One gene, *PRRG1*, was hypermethylated and downregulated, five genes (*MAP7D2, KDM6A, SMC1A, MED14, DDX3X)* were hypomethylated and upregulated, and seven genes (*SLC16A2, CA5B, CDK16, EIF1AX*, *XIST PUDP, PRKX)* were hypermethylated and upregulated (Fig .4). Two aDMR/DEG in blood were found in 47,XXY, vs. 46,XY (*DIP2C, DLGAP2*) and nine aDMR/DEGs were present in 45,X vs. 46,XX (*ACR, H3C6, RBBP8, ZSWIM5, NBR1, EBF4, MAML2, QTRT1, RNF216)*. Comparing 45,X vs. 46,XX in fat, two xDMR/DEGs were identified (*XIST* and *KDM6A*) (Additional file 1: Fig. S15). In 47,XXY vs 46,XY, 11 xDMR/DEGs, annotated to six genes, were identified (*XIST, MCF2, PUDP, EIF1AX, GRIPAP1, RNF128*) (Additional file 1: Fig. S15).In fat, no aDMR/DEGs pairs were observed from any comparison (Additional file 1: Fig. S15). In muscle, four xDMR/DEG pairs, three genes, were found in 45,X vs. 46,XX (*XIST, KDM6A*, and *CHIC1*) (Additional file 1: Fig. S15). In 47,XXY vs 46,XY, 18 xDMR/DEG, 10 genes were identified (*XIST, KDM6A, FGD1, SLC16A2, RGN, AMER1, SMC1A, RBBP7, NHS* and *ACOT9)* (Additional file 1: Fig. S15). Autosomal comparison of 47,XXY vs. 46,XY revealed no aDMR/DEG (Additional file 1: Fig. S15). Only one gene, *PRKAG2* was both hypermethylated and downregulated in 45,X vs. 46,XX (Additional file 1: Fig. S15).Our analysis identified several DMR/DEG pairs of particular interest. *KDM6A*, identified as a xDMR/DEG in both 45,X vs. 46,XX and 47,XXY vs. 46,XY in both blood, fat and muscle, hypomethylated and upregulated in 47,XXY, while hypermethylated and downregulated in 45,X vs. 46,XX. *MCF2* was hypermethylated in 47,XXY vs. 46,XY, and hypomethylated in 45,X vs. 46,XX, across all three tissues affected in three large DMRs. Although only a DEG in fat tissue. *MID2* was both hypomethylated in 45,X vs. both 46,XX and 46,XY in blood and muscle, while only downregulated in blood. *MID2* encodes the midline-2 protein, which is a member of the tripartite motif TRIM family with three zinc-binding domains. *SLC16A2* was hypomethylated and upregulated in 45,X and vice versa in 47,XXY in both blood and muscle tissue. *CCNB3* was hypomethylated in 45,X vs. 46,XX and hypermethylated in 47,XXY vs. 46,XY, across all three tissues, and upregulated in 45,X vs. 46,XX in blood. The protein encoded by this gene belongs to the cyclin family, that plays an essential role in cell cycle control (12), proper spindle reorganization and restoration of the interphase nucleus (13). *Ccnb3* plays a regulatory role in maintenance of the meiotic arrest and in mouse oocytes (14).

References

1.Pathak GA, Silzer TK, Sun J, Zhou Z, Daniel AA, Johnson L, O'Bryant S, Phillips NR, Barber RC. Genome-Wide Methylation of Mild Cognitive Impairment in Mexican Americans Highlights Genes Involved in Synaptic Transport, Alzheimer's Disease-Precursor Phenotypes, and Metabolic Morbidities. J Alzheimers Dis. 2019;72(3):733-749.
2.Lokapally A, Neuhaus H, Herfurth J, Hollemann T. Interplay of TRIM2 E3 Ubiquitin Ligase and ALIX/ESCRT Complex: Control of Developmental Plasticity During Early Neurogenesis. Cells. 2020;9(7).
3.Oo ZM, Adlat S, Sah RK, Myint MZZ, Hayel F, Chen Y, Htoo H, Bah FB, Bahadar N, Chan MK, Zhang L, Feng X, Zheng Y. Brain transcriptome study through CRISPR/Cas9 mediated mouse Dip2c gene knock-out. Gene. 2020;758:144975.
4.C Yuen RK, Merico D, Bookman M, L Howe J, Thiruvahindrapuram B, Patel RV, Whitney J, Deflaux N, Bingham J, Wang Z, Pellecchia G, Buchanan JA, Walker S, Marshall CR, Uddin M, Zarrei M, Deneault E, D'Abate L, Chan AJS, Koyanagi S, Paton T, Pereira SL, Hoang N, Engchuan W, Higginbotham EJ, Ho K, Lamoureux S, Li W, MacDonald JR, Nalpathamkalam T, Sung WWL, Tsoi FJ, Wei J, Xu L, Tasse A-M, Kirby E, Van Etten W, Twigger S, Roberts W, Drmic I, Jilderda S, Modi BM, Kellam B, Szego M, Cytrynbaum C, Weksberg R, Zwaigenbaum L, Woodbury-Smith M, Brian J, Senman L, Iaboni A, Doyle-Thomas K, Thompson A, Chrysler C, Leef J, Savion-Lemieux T, Smith IM, Liu X, Nicolson R, Seifer V, Fedele A, Cook EH, Dager S, Estes A, Gallagher L, Malow BA, Parr JR, Spence SJ, Vorstman J, Frey BJ, Robinson JT, Strug LJ, Fernandez BA, Elsabbagh M, Carter MT, Hallmayer J, Knoppers BM, Anagnostou E, Szatmari P, Ring RH, Glazer D, Pletcher MT, Scherer SW. Whole genome sequencing resource identifies 18 new candidate genes for autism spectrum disorder. Nat Neurosci. 2017;20(4):602-611.
5.Yang L, Zhao S, Ma N, Liu L, Li D, Li X, Wang Z, Song X, Wang Y, Wang D. Novel DIP2C gene splicing variant in an individual with focal infantile epilepsy. American journal of medical genetics Part A. 2022;188(1):210-215.
6.Tu H, Zhang A, Fu X, Xu S, Bai X, Wang H, Gao J. SMPX Deficiency Causes Stereocilia Degeneration and Progressive Hearing Loss in CBA/CaJ Mice. Front Cell Dev Biol. 2021;9:750023.
7.Viuff MH, Stochholm K, Juul S, Gravholt CH. Disorders of the eye, ear, skin, and nervous system in women with Turner syndrome -a nationwide cohort study. Eur J Hum Genet. 2021.
8.Erdélyi LS, Mann WA, Morris-Rosendahl DJ, Groß U, Nagel M, Várnai P, Balla A, Hunyady L. Mutation in the V2 vasopressin receptor gene, AVPR2, causes nephrogenic syndrome of inappropriate diuresis. Kidney Int. 2015;88(5):1070-1078.
9.Belling K, Russo F, Jensen AB, Dalgaard MD, Westergaard D, Rajpert-De Meyts E, Skakkebæk NE, Juul A, Brunak S. Klinefelter syndrome comorbidities linked to increased X chromosome gene dosage and altered protein interactome activity. Human Molecular Genetics. 2017;26(7):1219-1229.
10.Skakkebæk A, Nielsen MM, Trolle C, Vang S, Hornshøj H, Hedegaard J, Wallentin M, Bojesen A, Hertz JM, Fedder J, Østergaard JR, Pedersen JS, Gravholt CH. DNA hypermethylation and differential gene expression associated with Klinefelter syndrome. Scientific reports. 2018;8(1):13740.
11.Simonetti L, Ferreira LGA, Vidi AC, de Souza JS, Kunii IS, Melaragno MI, de Mello CB, Carvalheira G, Dias da Silva MR. Intelligence Quotient Variability in Klinefelter Syndrome Is Associated With GTPBP6 Expression Under Regulation of X-Chromosome Inactivation Pattern. Front Genet. 2021;12:724625.

12.Gallant P, Nigg EA. Identification of a novel vertebrate cyclin: cyclin B3 shares properties with both A- and B-type cyclins. EMBO J. 1994;13(3):595-605.
13.Zhang T, Qi ST, Huang L, Ma XS, Ouyang YC, Hou Y, Shen W, Schatten H, Sun QY. Cyclin B3 controls anaphase onset independent of spindle assembly checkpoint in meiotic oocytes. Cell Cycle. 2015;14(16):2648-2654.
14.Meng TG, Lei WL, Li J, Wang F, Zhao ZH, Li A, Wang ZB, Sun QY, Ou XH. Degradation of Ccnb3 is essential for maintenance of MII arrest in oocyte. Biochem Biophys Res Commun. 2020;521(1):265-269.
